# *Coelosynapha*, a new genus of the subfamily Gnoristinae (Diptera: Mycetophilidae) with a circumpolar, Holarctic distribution

**DOI:** 10.3897/BDJ.8.e54834

**Published:** 2020-09-10

**Authors:** Jostein Kjærandsen, Alexei Polevoi, Jukka Salmela

**Affiliations:** 1 Tromsø University Museum, UiT – The Arctic University of Norway, Tromsø, Norway Tromsø University Museum, UiT – The Arctic University of Norway Tromsø Norway; 2 Forest Research Institute of Karelian Research Centre of the Russian Academy of Sciences, Petrozavodsk, Russia Forest Research Institute of Karelian Research Centre of the Russian Academy of Sciences Petrozavodsk Russia; 3 Regional Museum of Lapland, Rovaniemi, Finland Regional Museum of Lapland Rovaniemi Finland; 4 Arctic Centre, University of Lapland, Rovaniemi, Finland Arctic Centre, University of Lapland Rovaniemi Finland

**Keywords:** *
Coelosynapha
*, Mycetophilidae, Gnoristinae, new genus, new species, DNA barcoding, Holarctic distribution, old-growth conifer forests.

## Abstract

**Background:**

The subfamily Gnoristinae is one of the most diverse and taxonomically difficult subfamilies of Mycetophilidae, with new species and genera being described almost every year from various parts of the world. Through inventories of fungus gnats in the Nordic Region and Russia, a genus and species new to science was discovered, yet with links back to an illustration made by the late French entomologist Loïc Matile in the 1980s. DNA barcoding aligned it with yet another species new to science, distributed across Canada and documented through The Barcode of Life Data System (BOLD) by Paul D. N. Hebert and colleagues at the BOLD team.

**New information:**

The new Holarctic genus, *Coelosynapha* **gen. n**. is described, consisting of two new species, the Palaearctic *Coelosynapha loici* **sp. n.** and the Nearctic *Coelosynapha heberti* **sp. n.** DNA-barcodes assign the two new species to distinctly separated (8.27% p-distance) Barcode Index Numbers (BINs) which are most closely aligned to unidentified species of Mycetophilidae from South Australia and Costa Rica on BOLD. The new genus shows morphological characteristics in between the two Holarctic genera *Coelosia* Winnertz, 1864 and *Synapha* Meigen, 1818 and further shows affinity to the southern continents genus *Austrosynapha* Tonnoir, 1929. The Palaearctic *Coelosynapha loici* **sp. n.**, for which habitat requirements are best documented, is largely restricted to pristine, old-growth conifer (mostly spruce, Picea abies ssp. obovata) forests within the boreal vegetation zone, although it is also recorded from hummock tundra along the Anadyr River in Far East Russia.

## Introduction

According to Fontaine et al. (2012), the average shelf life between discovery and description of a new species is 21 years across different taxa and the longest average shelf life is documented for terrestrial animals discovered by professional taxonomists living in rich countries. Sitting on huge collections with hundreds of potentially new species to science in our Nordic museum collections, the authors of this paper are guilty of this misconduct. For higher taxa, like describing a new genus, we suspect the average time can be even longer, given the uncertainty in relating it to known taxa often coupled with the scarcity of specimens of high quality. Still, discovering and describing new genera from the comparatively well-known insect fauna of the Nordic Region (see, for example, Ronquist et al. 2020) is not commonplace anymore.

**Figure 1.**
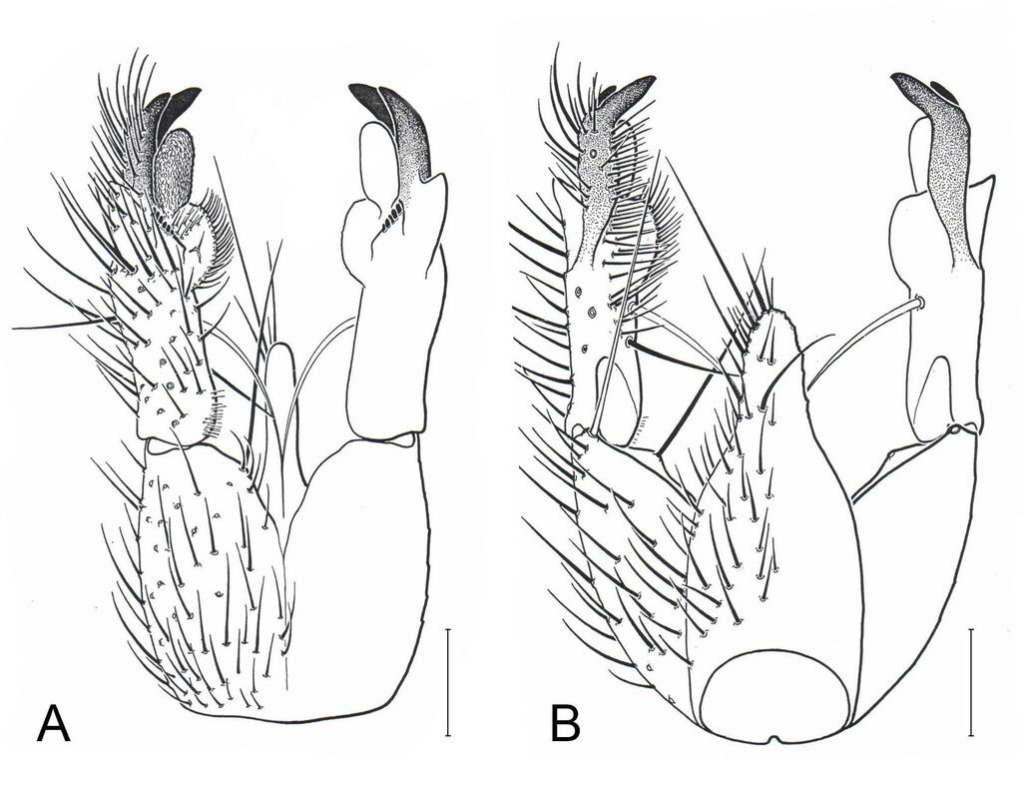
Some 25 year ago, the late French entomologist Loïc Matile (1938-2000) prepared these illustrations and a brief one-page description of a potential new genus based on a single specimen collected in Finland (A = ventral view, B = dorsal view.)

The current case concerns an undescribed genus known from the high north of Fennoscandia, from the Altai mountains and Far East Chukotka in Russia and from across southern Canada, i.e. a circumpolar taxon. Some 25 years ago, Geir E. E. Søli at the Natural History Museum in Oslo was made aware of an enigmatic species belonging to the subfamily Gnoristinae of the family Mycetophilidae when the late French entomologist Loïc Matile (1938-2000, see Daugeron et al. 2002) sent him illustrations (Fig. 1) and a brief one-page description of a potential new genus, based on a single specimen collected in Finland. On the top of the description page, Loïc Matile had noted by hand: “*Had this for years… What do you think? LM*”. Due to the scarce material, the case was shelved until the species was collected again, in 2009, this time also in Finnish Lapland by one of the authors (JS). Specimens were this time sent to a specialist in the USA, but again shelved without further progress. Further materials appeared from across Russia and Northern Norway. Meanwhile, after gathering at the 8^th^ International Congress of Dipterology in Germany in 2014, the authors of this paper started tighter cooperation on the discovery of new and problematic species of fungus gnats in the Nordic Region through a forum page on the Vibrant ScratchPad site “*Fungus Gnats Online*” (Fungus_Gnats_Online 2020). We all run national projects to record and describe the Nordic and Russian fauna of fungus gnats (see, for example, Jakovlev et al. 2014, Kjærandsen 2020, Kjærandsen et al. 2007b, Polevoi 2010, Polevoi and Barkalov 2017, Salmela et al. 2016, [Bibr B5815265][Bibr B5823099], Kjærandsen and Jordal 2007) and we are heavily engaged in DNA barcoding through the NorBOL (NorBOL 2020, Kjærandsen 2017) and FinBOL (FinBOL 2020) initiatives. Eventually, specimens of the enigmatic new species were obtained from several, mainly old-growth, coniferous sites across the Palaearctic Taiga, ranging from Reisa National Park in Norway in the west all the way to Chukotka in the Far East of Russia.

Specimens of the new taxon from Fennoscandia and Russia were submitted for barcoding and their automated Barcode Index Number (BIN) (Ratnasingham and Hebert 2013) assigned on The Barcode of Life Data System, BOLD (Ratnasingham and Hebert 2007) aligned them as the nearest neighbour to another unidentified and very similar species, sampled across southern Canada and barcoded through the Canadian Barcode of Life initiative (Hebert et al. 2016). Upon request to the Centre for Biodiversity Genomics, we were kindly offered a loan of the voucher material of this species, which, together with our Palaearctic material, are argued here to represent a new genus of the subfamily Gnoristinae, family Mycetophilidae.

The subfamily Gnoristinae appears to be amongst the most difficult branches of the Mycetophilidae to classify. Phylogenies (e.g. Kaspřák et al. 2018) are still rendering it paraphyletic with respect to Mycetophilinae and, according to Kaspřák et al. (2018), the Gnoristinae is one of the most diverse and taxonomically difficult subfamilies of Mycetophilidae, with new species and genera being described almost every year. Highly variable taxa have led to numerous small genera with few species being segregated, as well as species-rich, polyphyletic "trash bin" genera like *Dziedzickia* Johannsen, 1909. Within these genera, the variation in the classical Meigen-Winnertzian character system, which is largely based on wing venation, tends to break down, especially when tropical taxa are considered (see further discussion in Ševčík and Kjærandsen 2012).

## Materials and methods

### Specimen preparation and storage

**Figure 2.**
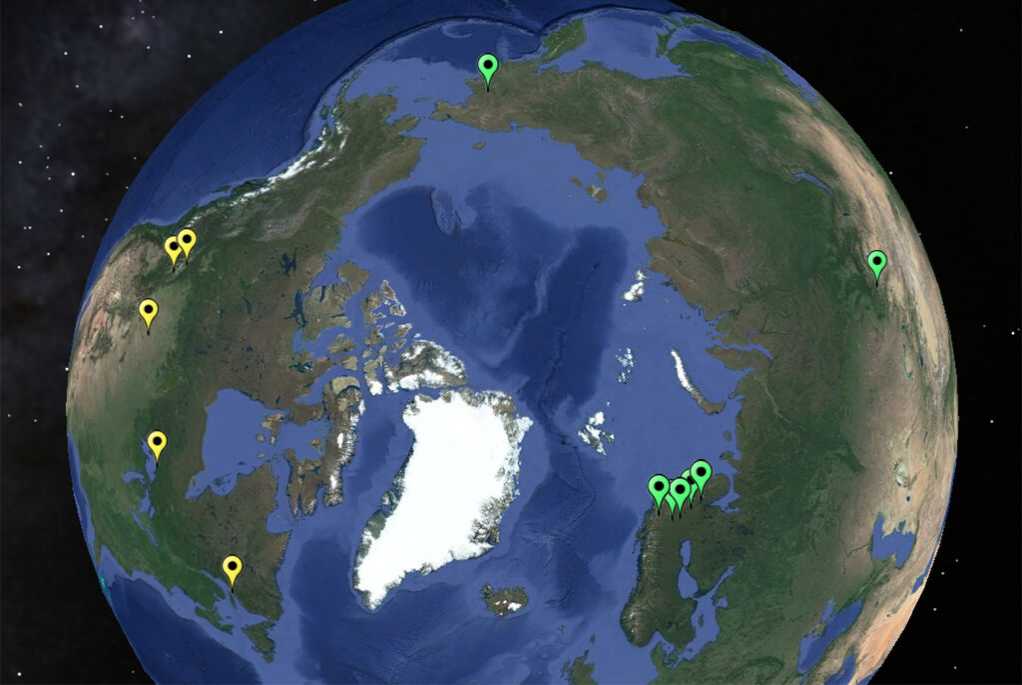
The circumpolar distribution of *Coelosynapha* gen. n. visualised on Google Earth. Green plots across the Palaearctic region display records of *Coelosynapha loici* sp. n., yellow plots across the Nearctic region display records of *Coelosynapha heberti* sp. n.

The studied specimens were collected over a period from 2009 to 2016 from 12 localities in North America and Eurasia (Fig. 2). Being initially stored in 70-95% ethanol, they were partly dried through baths of hexamethyldisilazane (HMDS, see Brown 1993) and pinned during the study. Terminalia were detached from the abdomen and treated by standard methods (macerated either in warm lactic acid or in a solution of potassium hydroxide (KOH), cleaned in distilled water and neutralised in acetic acid). After detailed study and imaging, the terminalia were placed into microvials with glycerine and pinned together with the rest of the specimen. The poor (fragmented) quality of the voucher materials borrowed from the Centre for Biodiversity Genomics did not allow for dry pinning, so the primary types from Canada were transferred from alcohol to glycerine in microvials on pins. Materials are deposited in the collections of the following institutions: Centre for Biodiversity Genomics, University of Guelph, Canada; California State Collection of Arthropods, Sacramento, California, USA; Tromsø University Museum, Tromsø, Norway; Regional Museum of Lapland, Rovaniemi, Finland; Siberian Zoological Museum, Novosibirsk, Russia; Tomsk State University, Tomsk, Russia; Forest Research Institute, Petrozavodsk, Russia.

### DNA barcoding

The 658 bp fragment of the mitochondrial protein-encoding cytochrome c oxidase subunit I (COI) was sequenced from a total of 10 *Coelosynapha loici* sp. n. specimens and five *C. heberti* sp. n. specimens. One leg from each fresh specimen was sent to the Canadian Centre for DNA barcoding, BIO (Guelph, Ontario, Canada), for DNA extraction and bi-directional Sanger sequencing as a part of the Norwegian Barcode of Life (NorBOL) (see Kjærandsen 2017) and Finnish Barcode of Life (FinBOL) initiatives, both branches of the International Barcode of Life project (iBOL). The new sequences are available from The Barcode of Life Data System (BOLD) and also as supplementary material (Suppl. material 1).

### Illustrations

A Leica MC170HD microscope camera, mounted on a Leica M205C stereomicroscope, was used to capture images of whole specimens and of detached terminalia macerated in hot lactic acid and stored in glycerine. Stacked images, merged for extended focus applying the Helicon Focus software, were subsequently moderately photo-shopped into illustrative plates. Digital illustrations (Fig. 10) were made with Inkscape vector drawing editor (http://inkscape.org).

### Terminology

The general terminology of body, wings and terminalia follows Søli (2017) with a few additional, more specific terms from Söli (1997).

## Taxon treatments

### Coelosynapha

Kjaerandsen, Polevoi & Salmela, 2020 gen. n.

93512EDB-7182-5834-AD3C-B1C94E6A787E

278C1F57-07CE-473E-A485-D10BD7925D82

Coelosynapha Coelosynapha loici Kjaerandsen, Polevoi & Salmela Status: new species described in this paper.

#### Description

**Figure 5.**
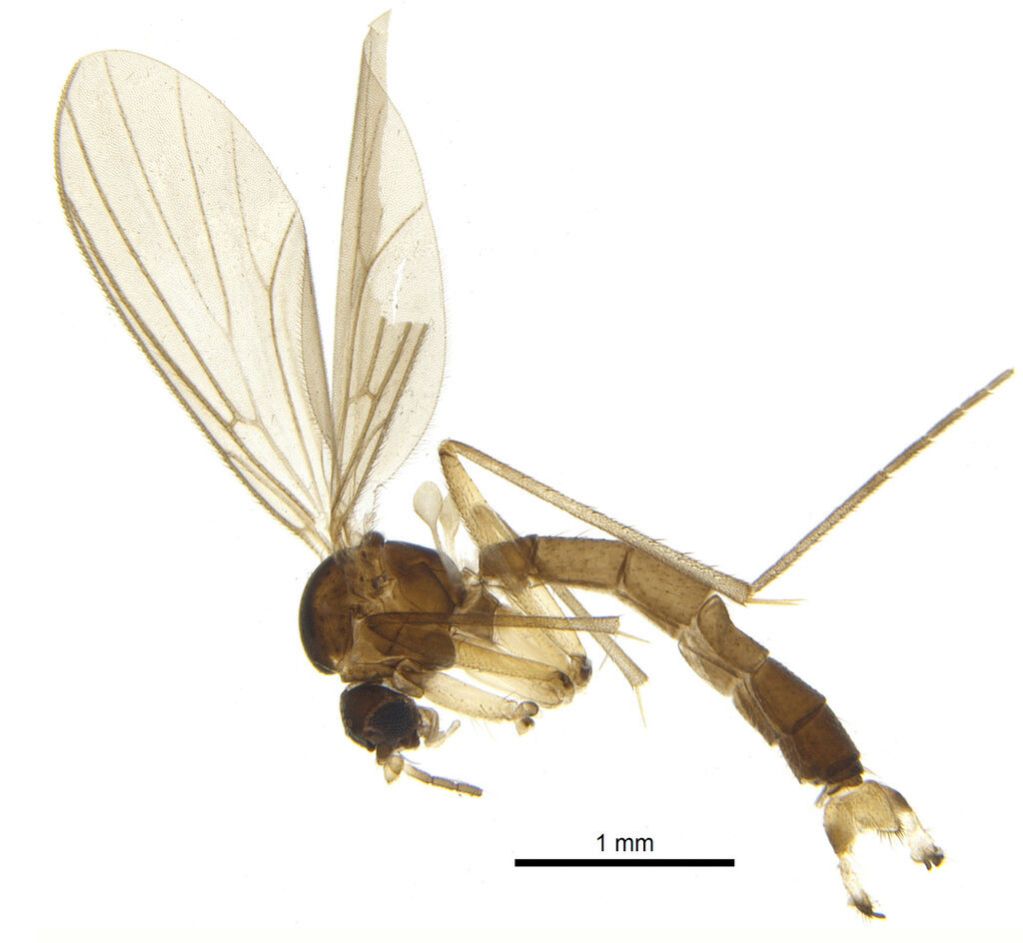
Holotype of *Coelosynapha heberti* sp. n., in 95% ethanol (CC-0, CBG Photography Group, Centre for Biodiversity Genomics).

A Gnoristinae genus, as presently known with moderately slender and quite small, down to 3 mm body length, species (Figs 3, 5). Colouration uniformly brown on head and body, darker preterminal segments, mostly yellow on legs and terminalia. Head (Fig. 4) round, eyes kidney-shaped with tendency of dorsal eye-bridge expansion (like in *Synapha* Meigen, 1818, unlike in *Coelosia* Winnertz, 1864 and *Austrosynapha* Tonnoir, 1929), interommatidia pubescent. Antenna moderately slender, with 16 segments, large, semi-globular pedicel and flagellar segments 2-3 times as long as wide (shorter in *Synapha*, distinctly longer in *Coelosia* and *Austrosynapha*). Mouth parts average, with five, gradually longer palpal segments, no clear sensory pit discernible in third segment (without slide mounting). Clypeus bud-shaped, shorter than face. Three ocelli in a near straight line (Fig. 4b, c), lateral ocellus more than two times its diameter from eye. Antepronotum with pair of strong antepronotal setae arching over the head (Fig. 4a, c). Mesonotum with setae in acrostichal and dorsocentral rows, devoid of setulae in between but rich in setae laterally (Fig. 4c). Meso- and metapleurites all without setae (Figs 4a, 5). Wings (Figs 6, 7) hyaline, unpatterned, wing membrane with irregularly arranged microtrichia. Costa produced more than half way between **R_4+5_** and **M_1_**, subcosta long, ending in **C** proximal to crossvein **Rs** (Fig. 6a), usually without, occasionally with crossvein **sc–r** (Fig. 7d). Radial sector variable, usually with **R_2+3_** present (Fig. 6d), sometimes with **R_2_** and **R_3_** separate (Fig. 6b), occasionally with only crossvein **Rs** (Fig. 7b). Anterior fork with stem more than 2× longer than **r–m**. Posterior fork short, rather widely divergent. All veins anterior of **iCu** with setae on dorsal surface except for basal transversal crossvein **tb** and **M**-stem. Legs with irregularly arranged setulae. Fore tarsus subequal in length to fore tibia. No sense organ discernible on mid tibia.

**Figure 8.**
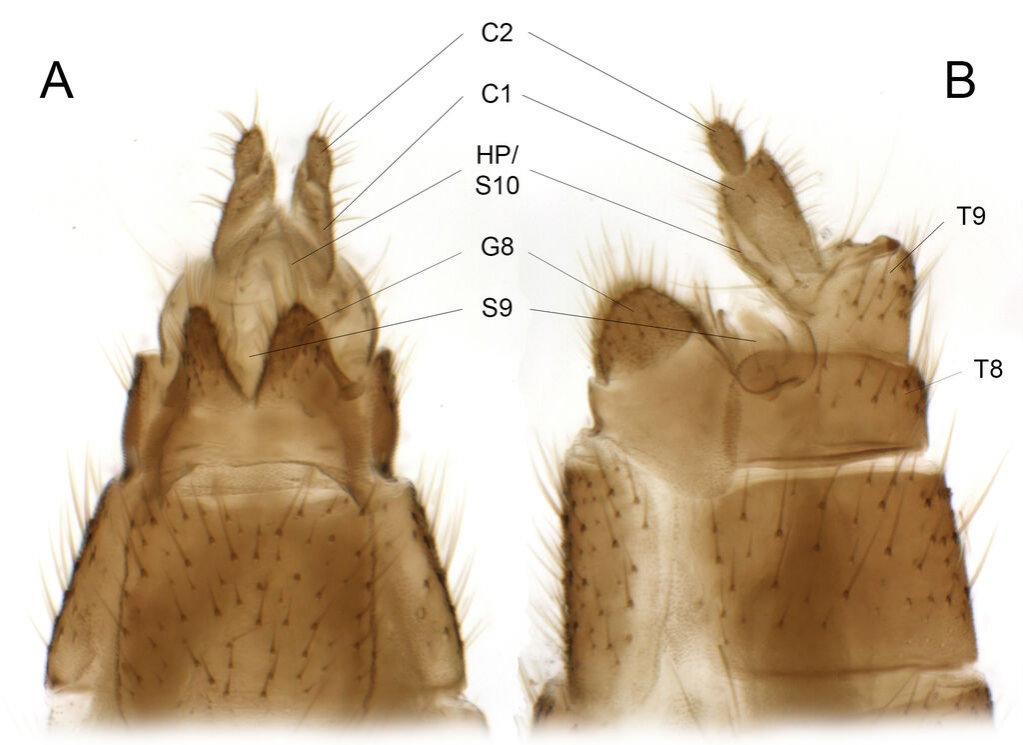
Terminalia of female paratype of *Coelosynapha heberti* sp. n. (A = dorsal view. B = lateral view). Abbreviations: **C1** = cercus 1; **C2** = cercus 2; **G8** = gonocoxite 8; **HP/S10** = hypoproct and sternite 10; **S9** = sternite 9; **T8** = tergite 8; **T9** = tergite 9.

Female terminalia (Fig. 8) rather truncated, with only hypoproct/sternite 10 and cerci somewhat elongated. Tergite 8 short, wide rectangular. Tergite 9 wide, subrectangular, with some setae extending towards epiproct dorsally. Cercus with first segment more than 2× as long as wide, second segment small, ovate. Gonocoxite 8 moderately split ventrally, with free, sclerotised, semicircular lamellae. Sternite 9 small, retracted.

Male terminalia (Figs 9, 10) with tergite 9 long and apically tapered. Cerci and epiproct usually partly retracted under tergite 9, but can be exposed (Fig. 9a). Gonocoxite open, semicircular with deep ventral cleft (Fig. 10a). Gonostyles large, elongated, exposed, apically with three, sclerotised, blunt, digitate projections and pair of long setae on inner surface (Fig. 10b). Aedeagal apparatus inconspicuous, tiny, elongated, framed within a small, pentagonal parameral structure (Fig. 10a).

#### Diagnosis

A Gnoristinae genus similar to *Austrosynapha* Tonnoir, 1929, *Coelosia* Winnertz, 1864 and *Synapha* Meigen, 1818 in general appearance, but with very characteristic and unique male terminalia with three blunt, digitate processes apically on the gonostyles (Figs 9a, b, 10a, b). It can be separated from the three genera by the wing venation having the combination of 1) extension of **C** long, ending more than half way between **R_4+5_** and **M_1_** (Fig. 6a, like in *Coelosia*, shorter in *Austrosynapha* and *Synapha*); 2) **Sc** ending in **C** at level of **Rs** (Fig. 6a, shorter in *Austrosynapha* which is variable for this character), usually without, but occasionally with **sc–r** present (Fig. 7d, always absent in *Coelosia*); 3) anterior fork petiole more than 2× length of crossvein **r–m** (Fig. 6a, like in most *Austrosynapha* and all *Synapha*, unlike in *Coelosia*); 4) short and wide posterior fork (Fig. 6a, like in all *Coelosia* and some *Austrosynapha*, unlike in *Synapha* and some *Austrosynapha*).

#### Etymology

The generic name is feminine gender and put together by the two genus names *Coelosia* Winnertz, 1864 and *Synapha* Meigen, 1818, indicating the affinity to and intermediate position between those two genera.

#### Distribution

Records of the new genus display a circumpolar distribution pattern from Fennoscandia to Far East Russia in the Palaearctic Region and across Canada in the Nearctic Region (Fig. 2).

### Coelosynapha loici

Kjaerandsen, Polevoi & Salmela, 2020 sp. n.

76F34AEE-AEB4-5526-9F37-459FC37274D2

ADD0785

17C0E23A-F95B-440F-97D8-CAA0105FE566

#### Materials

- **Type status:** Holotype. **Occurrence:** catalogNumber: TSZD-JKJ-102389; recordedBy: J. Kjaerandsen; individualCount: 1; sex: male; lifeStage: imago; preparations: Pinned, HMDS-dried from ethanol; occurrenceID: urn:uuid:5d9ce586-8e3f-4c53-b57c-4ce8f09debc9; **Location:** locationID: N-TRI-0045; country: Norway; stateProvince: Troms og Finnmark; municipality: Nordreisa; locality: Nokinivat, Reisa NP btw Naustneset and Nedrefosshytta; verbatimElevation: 63 m; locationRemarks: MT 6-2016, pine forest; verbatimCoordinates: 69 19 38 N 21 57 18 E; decimalLatitude: 69.3273; decimalLongitude: 21.9551; geodeticDatum: WGS84; coordinateUncertaintyInMeters: 10 m; georeferenceProtocol: GPS; **Identification:** identifiedBy: J. Kjaerandsen; dateIdentified: Oct-07-2016; **Event:** samplingProtocol: Malaise trap; eventDate: Jul 16–Sep 16 2016; **Record Level:** collectionID: TMU-JKJ-COL-000389; institutionCode: TSZ; collectionCode: TSZD; ownerInstitutionCode: Tromso University Museum; basisOfRecord: PreservedSpecimen
- **Type status:** Paratype. **Occurrence:** catalogNumber: TSZD-JKJ-102390; recordedBy: J. Kjaerandsen; individualCount: 1; sex: male; lifeStage: imago; preparations: Pinned, HMDS-dried from ethanol, terminalia detached, cleared and stored in glycerine vial; occurrenceID: urn:uuid:b5f8e15a-36bb-488d-8487-db9c007600d0; **Location:** locationID: N-TRI-0045; country: Norway; stateProvince: Troms og Finnmark; municipality: Nordreisa; locality: Nokinivat, Reisa NP btw Naustneset and Nedrefosshytta; verbatimElevation: 63 m; locationRemarks: MT 6-2016, pine forest; verbatimCoordinates: 69 19 38 N 21 57 18 E; decimalLatitude: 69.3273; decimalLongitude: 21.9551; geodeticDatum: WGS84; coordinateUncertaintyInMeters: 10 m; georeferenceProtocol: GPS; **Identification:** identifiedBy: J. Kjaerandsen; dateIdentified: Oct-07-2016; **Event:** samplingProtocol: Malaise trap; eventDate: Jul 16–Sep 16 2016; **Record Level:** collectionID: TMU-JKJ-COL-000389; institutionCode: TSZ; collectionCode: TSZD; ownerInstitutionCode: Tromso University Museum; basisOfRecord: PreservedSpecimen
- **Type status:** Paratype. **Occurrence:** catalogNumber: TSZD-JKJ-102391; recordedBy: J. Kjaerandsen; individualCount: 1; sex: male; lifeStage: imago; preparations: Pinned, HMDS-dried from ethanol, terminalia detached, cleared and stored in glycerine vial; occurrenceID: urn:uuid:69b453cf-4f75-41e2-8667-41c5fa37eadd; **Location:** locationID: N-TRI-0045; country: Norway; stateProvince: Troms og Finnmark; municipality: Nordreisa; locality: Nokinivat, Reisa NP btw Naustneset and Nedrefosshytta; verbatimElevation: 63 m; locationRemarks: MT 6-2016, pine forest; verbatimCoordinates: 69 19 38 N 21 57 18 E; decimalLatitude: 69.3273; decimalLongitude: 21.9551; geodeticDatum: WGS84; coordinateUncertaintyInMeters: 10 m; georeferenceProtocol: GPS; **Identification:** identifiedBy: J. Kjaerandsen; dateIdentified: Oct-07-2016; **Event:** samplingProtocol: Malaise trap; eventDate: Jul 16–Sep 16 2016; **Record Level:** collectionID: TMU-JKJ-COL-000389; institutionCode: TSZ; collectionCode: TSZD; ownerInstitutionCode: Tromso University Museum; basisOfRecord: PreservedSpecimen
- **Type status:** Paratype. **Occurrence:** catalogNumber: TSZD-JKJ-102749; recordedBy: J. Kjaerandsen; individualCount: 1; sex: male; lifeStage: imago; preparations: Pinned, HMDS-dried from ethanol; occurrenceID: urn:uuid:4fe1af9c-e93a-4da7-8a17-1164022e4302; **Location:** locationID: N-TRI-0045; country: Norway; stateProvince: Troms og Finnmark; municipality: Nordreisa; locality: Nokinivat, Reisa NP btw Naustneset and Nedrefosshytta; verbatimElevation: 63 m; locationRemarks: MT 6-2016, pine forest; verbatimCoordinates: 69 19 38 N 21 57 18 E; decimalLatitude: 69.3273; decimalLongitude: 21.9551; geodeticDatum: WGS84; coordinateUncertaintyInMeters: 10 m; georeferenceProtocol: GPS; **Identification:** identifiedBy: J. Kjaerandsen; dateIdentified: Jan-06-2017; **Event:** samplingProtocol: Malaise trap; eventDate: Jul 16–Sep 16 2016; **Record Level:** collectionID: TMU-JKJ-COL-000389; institutionCode: TSZ; collectionCode: TSZD; ownerInstitutionCode: Tromso University Museum; basisOfRecord: PreservedSpecimen
- **Type status:** Paratype. **Occurrence:** catalogNumber: TSZD-JKJ-102750; recordedBy: J. Kjaerandsen; individualCount: 1; sex: male; lifeStage: imago; preparations: Pinned, HMDS-dried from ethanol; occurrenceID: urn:uuid:a01d62c3-4453-4d80-9924-1e66531a6e41; **Location:** locationID: N-TRI-0045; country: Norway; stateProvince: Troms og Finnmark; municipality: Nordreisa; locality: Nokinivat, Reisa NP btw Naustneset and Nedrefosshytta; verbatimElevation: 63 m; locationRemarks: MT 6-2016, pine forest; verbatimCoordinates: 69 19 38 N 21 57 18 E; decimalLatitude: 69.3273; decimalLongitude: 21.9551; geodeticDatum: WGS84; coordinateUncertaintyInMeters: 10 m; georeferenceProtocol: GPS; **Identification:** identifiedBy: J. Kjaerandsen; dateIdentified: Jan-06-2017; **Event:** samplingProtocol: Malaise trap; eventDate: Jul 16–Sep 16 2016; **Record Level:** collectionID: TMU-JKJ-COL-000389; institutionCode: TSZ; collectionCode: TSZD; ownerInstitutionCode: Tromso University Museum; basisOfRecord: PreservedSpecimen
- **Type status:** Paratype. **Occurrence:** catalogNumber: TSZD-JKJ-102751; recordedBy: J. Kjaerandsen; individualCount: 1; sex: male; lifeStage: imago; preparations: Pinned, HMDS-dried from ethanol; occurrenceID: urn:uuid:a01e5902-a3bd-4b97-8196-de178527d292; **Location:** locationID: N-TRI-0045; country: Norway; stateProvince: Troms og Finnmark; municipality: Nordreisa; locality: Nokinivat, Reisa NP btw Naustneset and Nedrefosshytta; verbatimElevation: 63 m; locationRemarks: MT 6-2016, pine forest; verbatimCoordinates: 69 19 38 N 21 57 18 E; decimalLatitude: 69.3273; decimalLongitude: 21.9551; geodeticDatum: WGS84; coordinateUncertaintyInMeters: 10 m; georeferenceProtocol: GPS; **Identification:** identifiedBy: J. Kjaerandsen; dateIdentified: Jan-06-2017; **Event:** samplingProtocol: Malaise trap; eventDate: Jul 16–Sep 16 2016; **Record Level:** collectionID: TMU-JKJ-COL-000389; institutionCode: TSZ; collectionCode: TSZD; ownerInstitutionCode: Tromso University Museum; basisOfRecord: PreservedSpecimen
- **Type status:** Paratype. **Occurrence:** catalogNumber: TSZD-JKJ-102752; recordedBy: J. Kjaerandsen; individualCount: 1; sex: male; lifeStage: imago; preparations: Pinned, HMDS-dried from ethanol; occurrenceID: urn:uuid:49a80161-b4e1-4619-affb-88c05a2b6ae8; **Location:** locationID: N-TRI-0045; country: Norway; stateProvince: Troms og Finnmark; municipality: Nordreisa; locality: Nokinivat, Reisa NP btw Naustneset and Nedrefosshytta; verbatimElevation: 63 m; locationRemarks: MT 6-2016, pine forest; verbatimCoordinates: 69 19 38 N 21 57 18 E; decimalLatitude: 69.3273; decimalLongitude: 21.9551; geodeticDatum: WGS84; coordinateUncertaintyInMeters: 10 m; georeferenceProtocol: GPS; **Identification:** identifiedBy: J. Kjaerandsen; dateIdentified: Jan-06-2017; **Event:** samplingProtocol: Malaise trap; eventDate: Jul 16–Sep 16 2016; **Record Level:** collectionID: TMU-JKJ-COL-000389; institutionCode: TSZ; collectionCode: TSZD; ownerInstitutionCode: Tromso University Museum; basisOfRecord: PreservedSpecimen
- **Type status:** Paratype. **Occurrence:** catalogNumber: TSZD-JKJ-102753; recordedBy: J. Kjaerandsen; individualCount: 1; sex: male; lifeStage: imago; preparations: Pinned, HMDS-dried from ethanol; occurrenceID: urn:uuid:6d2863e8-c396-4188-856d-fc3ea8d4575d; **Location:** locationID: N-TRI-0045; country: Norway; stateProvince: Troms og Finnmark; municipality: Nordreisa; locality: Nokinivat, Reisa NP btw Naustneset and Nedrefosshytta; verbatimElevation: 63 m; locationRemarks: MT 6-2016, pine forest; verbatimCoordinates: 69 19 38 N 21 57 18 E; decimalLatitude: 69.3273; decimalLongitude: 21.9551; geodeticDatum: WGS84; coordinateUncertaintyInMeters: 10 m; georeferenceProtocol: GPS; **Identification:** identifiedBy: J. Kjaerandsen; dateIdentified: Jan-06-2017; **Event:** samplingProtocol: Malaise trap; eventDate: Jul 16–Sep 16 2016; **Record Level:** collectionID: TMU-JKJ-COL-000389; institutionCode: TSZ; collectionCode: TSZD; ownerInstitutionCode: Tromso University Museum; basisOfRecord: PreservedSpecimen
- **Type status:** Other material. **Occurrence:** catalogNumber: TSZD-JKJ-111229; recordedBy: J. Kjaerandsen; individualCount: 1; sex: male; lifeStage: imago; preparations: In 80% alcohol, in freezer; occurrenceID: urn:uuid:00150511-d1f9-4d15-8c76-cf7c00a1c58d; **Location:** locationID: N-TRI-0045; country: Norway; stateProvince: Troms og Finnmark; municipality: Nordreisa; locality: Nokinivat, Reisa NP btw Naustneset and Nedrefosshytta; verbatimElevation: 63 m; locationRemarks: MT 6-2016, pine forest; verbatimCoordinates: 69 19 38 N 21 57 18 E; decimalLatitude: 69.3273; decimalLongitude: 21.9551; geodeticDatum: WGS84; coordinateUncertaintyInMeters: 10 m; georeferenceProtocol: GPS; **Identification:** identifiedBy: J. Kjaerandsen; dateIdentified: Feb-28-2020; **Event:** samplingProtocol: Malaise trap; eventDate: Jul 16–Sep 16 2016; **Record Level:** collectionID: TMU-JKJ-COL-000389; institutionCode: TSZ; collectionCode: TSZD; ownerInstitutionCode: Tromso University Museum; basisOfRecord: PreservedSpecimen
- **Type status:** Paratype. **Occurrence:** catalogNumber: MYCE-NV-2013-0230; recordedBy: J. Salmela; individualCount: 1; sex: male; lifeStage: imago; preparations: In 80% alcohol, terminalia detached, cleared and stored in glycerine vial; occurrenceID: urn:uuid:b0b3b9a8-f241-4219-848a-12bd65866d97; **Location:** country: Finland; stateProvince: Lapponia kemensis pars occidentalis; municipality: Kittilä; locality: Pomokaira, Tarpomapää; verbatimElevation: 300 m; locationRemarks: Old-growth spruce forest; decimalLatitude: 67.8205; decimalLongitude: 25.9192; geodeticDatum: WGS84; coordinateUncertaintyInMeters: 10 m; georeferenceProtocol: GPS; **Identification:** identifiedBy: J. Salmela; **Event:** samplingProtocol: Malaise trap; eventDate: June 1–29 2009; **Record Level:** collectionID: MYCE-NV-2013-0230; institutionCode: LMM; collectionCode: LMM; ownerInstitutionCode: Regional Museum of Lapland; basisOfRecord: PreservedSpecimen
- **Type status:** Paratype. **Occurrence:** recordedBy: J. Salmela; individualCount: 1; sex: male; lifeStage: imago; preparations: In 80% alcohol; occurrenceID: urn:uuid:98e5f84a-192c-4750-bf98-ddbe1006bf4f; **Location:** country: Finland; stateProvince: Lapponia kemensis pars occidentalis; municipality: Kittilä; locality: Pomokaira, Tarpomapää; verbatimElevation: 300 m; locationRemarks: Old-growth spruce forest; decimalLatitude: 67.8205; decimalLongitude: 25.9192; geodeticDatum: WGS84; coordinateUncertaintyInMeters: 10 m; georeferenceProtocol: GPS; **Identification:** identifiedBy: J. Salmela; **Event:** samplingProtocol: Malaise trap; eventDate: June 1–29 2009; **Record Level:** institutionCode: CSCA; collectionCode: CSCA; ownerInstitutionCode: California State Collection of Arthropods; basisOfRecord: PreservedSpecimen
- **Type status:** Paratype. **Occurrence:** catalogNumber: DIPT-JS-2015-0033; recordedBy: J. Salmela; individualCount: 1; sex: male; lifeStage: imago; preparations: In 80% alcohol; occurrenceID: urn:uuid:3fb6eaa2-3d60-4750-93e7-d0dbbdf55b49; **Location:** country: Finland; stateProvince: Lapponia enontekioensis; municipality: Enontekiö; locality: Pallas-Yllästunturi National Park, Röyninkuru; verbatimElevation: 360 m; locationRemarks: Old-growth spruce forest, herb-rich; decimalLatitude: 68.1482; decimalLongitude: 24.075; geodeticDatum: WGS84; coordinateUncertaintyInMeters: 10 m; georeferenceProtocol: GPS; **Identification:** identifiedBy: J. Salmela; **Event:** samplingProtocol: Malaise trap; eventDate: Jul 5–Aug 7 2013; **Record Level:** collectionID: DIPT-JS-2015-0033; institutionCode: LMM; collectionCode: LMM; ownerInstitutionCode: Regional Museum of Lapland; basisOfRecord: PreservedSpecimen
- **Type status:** Paratype. **Occurrence:** catalogNumber: DIPT-JS-2014-0030; recordedBy: J. Salmela; individualCount: 1; sex: male; lifeStage: imago; preparations: In 80% alcohol; occurrenceID: urn:uuid:cd2dc33f-5eeb-4318-8067-e3b856374ff8; **Location:** country: Finland; stateProvince: Regio kuusamoensis; municipality: Salla; locality: Värriö Strict Nature Reserve, Kuntasjoki; verbatimElevation: 330 m; locationRemarks: Old-growth spruce forest, herb-rich; decimalLatitude: 67.7494; decimalLongitude: 29.6169; geodeticDatum: WGS84; coordinateUncertaintyInMeters: 10 m; georeferenceProtocol: GPS; **Identification:** identifiedBy: J. Salmela; **Event:** samplingProtocol: Malaise trap; eventDate: Jun 29–Jul 29 2013; **Record Level:** collectionID: DIPT-JS-2014-0030; institutionCode: LMM; collectionCode: LMM; ownerInstitutionCode: Regional Museum of Lapland; basisOfRecord: PreservedSpecimen
- **Type status:** Paratype. **Occurrence:** catalogNumber: DIPT-JS-2015-0151; recordedBy: J. Salmela; individualCount: 1; sex: male; lifeStage: imago; preparations: In 80% alcohol; occurrenceID: urn:uuid:25fc9a52-bb58-4f8c-8244-1f6437742075; **Location:** country: Finland; stateProvince: Lapponia enontekioensis; municipality: Enontekiö; locality: Pallas-Yllästunturi National Park, Röyninkuru; verbatimElevation: 360 m; locationRemarks: Old-growth spruce forest, herb-rich; decimalLatitude: 68.1482; decimalLongitude: 24.075; geodeticDatum: WGS84; coordinateUncertaintyInMeters: 10 m; georeferenceProtocol: GPS; **Identification:** identifiedBy: J. Salmela; **Event:** samplingProtocol: Malaise trap; eventDate: Jul 5–Aug 7 2013; **Record Level:** collectionID: DIPT-JS-2015-0151; institutionCode: LMM; collectionCode: LMM; ownerInstitutionCode: Regional Museum of Lapland; basisOfRecord: PreservedSpecimen
- **Type status:** Other material. **Occurrence:** catalogNumber: DIPT-JS-2015-0393; recordedBy: J. Salmela; individualCount: 1; sex: male; lifeStage: imago; preparations: In 80% alcohol; **Location:** country: Finland; stateProvince: Lapponia kemensis pars orientalis; municipality: Savukoski; locality: Ainijärvi; verbatimElevation: 260 m; locationRemarks: Old-growth spruce forest, herb-rich; decimalLatitude: 67.7701; decimalLongitude: 29.4332; geodeticDatum: WGS84; coordinateUncertaintyInMeters: 10 m; georeferenceProtocol: GPS; **Identification:** identifiedBy: J. Salmela; **Event:** samplingProtocol: Malaise trap; eventDate: Jul 30–Sep 28 2015; **Record Level:** collectionID: DIPT-JS-2015-0393; institutionCode: LMM; collectionCode: LMM; ownerInstitutionCode: Regional Museum of Lapland; basisOfRecord: PreservedSpecimen
- **Type status:** Paratype. **Occurrence:** catalogNumber: DIPT-JS-2016-0197; recordedBy: J. Salmela; individualCount: 1; sex: male; lifeStage: imago; preparations: In 80% alcohol; **Location:** country: Finland; stateProvince: Lapponia kemensis pars orientalis; municipality: Savukoski; locality: Ainijärvi; verbatimElevation: 260 m; locationRemarks: Old-growth spruce forest; decimalLatitude: 67.7688; decimalLongitude: 29.4286; geodeticDatum: WGS84; coordinateUncertaintyInMeters: 10 m; georeferenceProtocol: GPS; **Identification:** identifiedBy: J. Salmela; **Event:** samplingProtocol: Malaise trap; eventDate: Jul 30–Sep 28 2015; **Record Level:** collectionID: DIPT-JS-2016-0197; institutionCode: LMM; collectionCode: LMM; ownerInstitutionCode: Regional Museum of Lapland; basisOfRecord: PreservedSpecimen
- **Type status:** Other material. **Occurrence:** catalogNumber: DIPT-JS-2016-0599; recordedBy: J. Salmela; individualCount: 1; sex: male; lifeStage: imago; preparations: In 80% alcohol; **Location:** country: Finland; stateProvince: Lapponia kemensis pars orientalis; municipality: Savukoski; locality: Ainijärvi; verbatimElevation: 260 m; locationRemarks: Old-growth spruce forest; decimalLatitude: 67.7688; decimalLongitude: 29.4286; geodeticDatum: WGS84; coordinateUncertaintyInMeters: 10 m; georeferenceProtocol: GPS; **Identification:** identifiedBy: J. Salmela; **Event:** samplingProtocol: Malaise trap; eventDate: Jun 30–Jul 25 2015; **Record Level:** collectionID: DIPT-JS-2016-0599; institutionCode: LMM; collectionCode: LMM; ownerInstitutionCode: Regional Museum of Lapland; basisOfRecord: PreservedSpecimen
- **Type status:** Other material. **Occurrence:** recordedBy: A. Polevoi; individualCount: 1; sex: male; lifeStage: imago; preparations: Pinned, terminalia detached, cleared and stored in glycerine vial; occurrenceID: urn:uuid:2db6d72a-a309-406d-8917-6e1ee5e2f15d; **Location:** country: Russia; stateProvince: Murmansk Province; locality: Laplandskiy Nature Reserve, Lisiy Ruchei; verbatimElevation: 237 m; decimalLatitude: 67.6512; decimalLongitude: 32.5985; geodeticDatum: WGS84; coordinateUncertaintyInMeters: 10 m; georeferenceProtocol: GPS; **Identification:** identifiedBy: A. Polevoi; **Event:** samplingProtocol: Malaise trap; eventDate: May 28–Sep 20 2014; **Record Level:** institutionCode: FRIP; ownerInstitutionCode: Forest Research Institute KRC RAS; basisOfRecord: PreservedSpecimen
- **Type status:** Paratype. **Occurrence:** recordedBy: A. Barkalov; individualCount: 1; sex: male; lifeStage: imago; preparations: Pinned, terminalia detached, cleared and stored in glycerine vial; occurrenceID: urn:uuid:a388b08d-4177-443f-be4e-1aa92cfe4077; **Location:** country: Russia; stateProvince: Chukotka Autonomyus District; locality: Anadyr r., 30 km lower l. Krasnoe; verbatimElevation: 15 m; decimalLatitude: 64.72; decimalLongitude: 175.21; geodeticDatum: WGS84; coordinateUncertaintyInMeters: 1 km; **Identification:** identifiedBy: A. Polevoi; **Event:** samplingProtocol: Yellow pan trap; eventDate: Jun 25–Jul 19 2014; **Record Level:** institutionCode: SZM; ownerInstitutionCode: Siberian Zoological Museum; basisOfRecord: PreservedSpecimen
- **Type status:** Other material. **Occurrence:** recordedBy: Ju. Timchuk; individualCount: 2; sex: male; lifeStage: imago; preparations: In 80% alcohol, terminalia detached, cleared and stored in glycerine vial; occurrenceID: urn:uuid:b8160746-46c3-4fdc-94ea-d98b0ce9064f; **Location:** country: Russia; stateProvince: Republic of Altai; locality: 7 km NNE of Kurai; verbatimElevation: 2144 m; decimalLatitude: 50.2957; decimalLongitude: 87.902; geodeticDatum: WGS84; coordinateUncertaintyInMeters: 10 m; georeferenceProtocol: GPS; **Identification:** identifiedBy: A. Polevoi; **Event:** samplingProtocol: Window trap; eventDate: May 28–Sep 20 2014; **Record Level:** institutionCode: UTR; ownerInstitutionCode: Tomsk State University; basisOfRecord: PreservedSpecimen

#### Description

**Male**. (Figs 3, 4, 6, 9, 10, 11a, b, 12, *n* = 8)

Head (Fig. 4) dark brown, vertex sparsely covered by short, light hairs. Three ocelli almost in line, lateral ocellus placed 2.5 to 3 times its diameter from eye margin. Clypeus bud-shaped, 2x wider than long. Palpi 5-segmented, yellowish-brown. First three segments short, fifth segment 1.75–1.5 as long as fourth segment. Antenna with scape and pedicel usually largely brown, sometimes yellowish. Pedicel with outstanding apico-dorsal seta about as long as first flagellomere. Flagellomeres bearing light, curved, decumbent setulae, their length not exceeding width of respective flagellomeres.

Thorax (Fig. 4a, c). Scutum usually dark-brown (with yellow humeral area in Chukotka specimen), thinly dusted, with yellow setulae. Pleural sclerites glabrous, dark brown, antepronotum and proepisternum yellowish. Laterotergite and mediotergite bare. Strong antepronotal seta about as long as head. Scutellum with two strong setae and four small setae.

Wings (Fig. 6). Wing length 2.6–2.9 mm. **Sc** ending in **C**, cross-vein **sc–r** absent. **C** produced beyond apex of R5 for about 2/3 of the distance between **R_4+5_** and **M_1_, R_2+3_** present, forming rectangular cell, occasionally separate veins **R_2_** and **R_3_** developed. Anterior fork long, divergent. Posterior fork short, its long stem is forked well beyond point of furcation of anterior fork. Veins **M_4_** and **CuA** rather widely divergent. **Sc** with 3-5 macrotrichia on apical half. Basal transversal crossvein (**tb**) and **M**-stem bare, all other veins anterior of **iCu** fold setose. Halters pale yellowish.

Legs. Legs yellow with a brown tinge on trochanter, tibia and tarsi. Fore tarsus 1 slightly longer than fore tibia (ratio 1.06 in holotype).

Abdomen. Abdominal tergites and sternites brown, bearing light hairs. Tergites 7 and 8 rudimentary in size, sternite 8 lingulate.

Terminalia (Figs 9, 10, 11a, b, 12). Yellowish-brown with pale yellow gonostyles except on sclerotised, blackish apical processes, setation mostly pale. Tergite 9 elongated, narrowed to half width on apical half, apex rounded, bearing 12-15 apico-ventral, stout setae. Cerci and hypoproct/sternite 10 partly retracted in under tergite 9, expandable. Gonocoxites with a deep ventral cleft. Gonocoxite with a hyaline, apico-mesal, long seta, almost as long as gonostylus. Gonostylus elongated, with three blunt, digitate projections apically (marked as a, b & c in Fig. 12a, c), three lamellae subapically (marked as d, e & f in Fig. 12a, b, c) and two long bristles mesally (marked as g & h in Fig. 12b, c). Apical projection (a) finger-like, apically rounded, widest medially and bent dorsally. Subapical projection (b) wide, black, apically rounded, glabrous. Basal projection (c) black, hollow, having reticulate surface pattern, glabrous, apically rounded. Lateral lamella (d) triangular, high and narrow, glabrous. Ventral lamella (e) a sharp low ridge, edged with 6 blunt teeth-shaped setae. Ventromesal lamella (f) forming a blunt ridge densely covered with setulae.

Aedeagus inconspicuous, parameres fused.

**Female** unknown.

#### Etymology

*Coelosynapha loici* sp. n. is named in honour of the late French entomologist Loïc Matile (1938–2000) who first studied the new species and illustrated its terminalia (Fig. 1) way back in the mid 1980s. It is further an expression of the importance of classical morphological studies including detailed illustrations to be retained in the new integrative science of taxonomy.

#### Distribution

The new species has a wide Palaearctic range in boreo-mountainous localities, it has been collected from the High North boreal forest of Fennoscandia (Norway, Finland, NW Russia), from a high mountain site in Asian part of Russia and from hummock tundra in Far East Russia, at a total of seven localities (Fig. 2).

#### Ecology

Six of the collecting sites are within the boreal vegetation zone while the record from Chukotka is from the low arctic ecoregion. The European sites, one in Norway, three in Finland, one in Russia, are all northern boreal, close to the range limit of spruce (Picea abies ssp. obovata), lying some 140-180 km north of the arctic circle. All European collecting sites are pristine, boreal old-growth forests, dominated by spruce or occasionally pine (Reisadalen NP) and with scattered deciduous trees (birch, *Betula pubescens* and goat willow, *Salix caprea*). The ground layer is characterised by mosses, especially *Pleurozium schreberi* and bilberry (*Vaccinium myrtillus*). Malaise traps on these sites have been set in the vicinity of springs or cold headwater streams. The specimens from Altai were collected at an altitude of 2144 m above sea level in steppe type *Larix* forest. The Chukotka specimen was collected with a yellow pan trap set in hummock tundra along the Anadyr river at 5-10 m above sea level.

### Coelosynapha heberti

Kjaerandsen, Polevoi & Salmela, 2020 sp. n.

CE03FDDF-4C7D-54C0-A896-5CD9ED45DA8F

ACI5160

090406C0-6C4D-4CF9-8A25-5CDB1FF07186

#### Materials

- **Type status:** Holotype. **Occurrence:** catalogNumber: BIOUG10047-B08; recordNumber: GMP#01494; recordedBy: Cavan Harpur; individualCount: 1; sex: male; lifeStage: imago; preparations: Pinned, whole specimen in glycerine vial, terminalia detached, cleared and stored in separate glycerine vial; associatedMedia: http://www.boldsystems.org/index.php/MAS_DataRetrieval_OpenSpecimen?selectedrecordid=4186616; occurrenceID: urn:uuid:bf70f2b5-796a-420c-b4a9-9861e66ebab9; **Location:** country: Canada; stateProvince: Ontario; locality: Pukaskwa National Park,near Park Office; decimalLatitude: 48.601; decimalLongitude: -86.2893; geodeticDatum: WGS84; **Identification:** identifiedBy: Jostein Kjaerandsen; dateIdentified: Feb-28-2020; **Event:** eventDate: Jun 24–Jul 8 2013; **Record Level:** institutionCode: CBG; collectionCode: BIOUG; ownerInstitutionCode: Centre for Biodiversity Genomics; basisOfRecord: PreservedSpecimen
- **Type status:** Paratype. **Occurrence:** catalogNumber: BIOUG12690-F07; recordNumber: GMP#01116; recordedBy: Park Staff; individualCount: 1; sex: female; lifeStage: imago; preparations: Pinned, whole specimen in glycerine vial, terminalia detached, cleared and stored in separate glycerine vial; associatedMedia: http://www.boldsystems.org/index.php/MAS_DataRetrieval_OpenSpecimen?selectedrecordid=4487356; occurrenceID: urn:uuid:5b79b318-2c59-448d-9ead-87c458abf1a7; **Location:** country: Canada; stateProvince: Quebec; locality: Mingan Archipelago National Park Reserve; decimalLatitude: 50.2135; decimalLongitude: -63.7979; geodeticDatum: WGS84; **Identification:** identifiedBy: Jostein Kjaerandsen; dateIdentified: Feb-28-2020; **Event:** eventDate: 12–18 Sep 2013; **Record Level:** institutionCode: CBG; collectionCode: BIOUG; ownerInstitutionCode: Centre for Biodiversity Genomics; basisOfRecord: PreservedSpecimen
- **Type status:** Paratype. **Occurrence:** catalogNumber: BIOUG19436-E05; recordNumber: GMP#04291; recordedBy: Jamie; individualCount: 1; sex: female; lifeStage: imago; preparations: In 95% alcohol; associatedMedia: http://www.boldsystems.org/index.php/MAS_DataRetrieval_OpenSpecimen?selectedrecordid=5534257; occurrenceID: urn:uuid:aab42085-0f79-4ee4-9035-d31e6f488f36; **Location:** country: Canada; stateProvince: British Columbia; locality: Bunkhouse; decimalLatitude: 51.365; decimalLongitude: -116.528; geodeticDatum: WGS84; **Identification:** identifiedBy: Jostein Kjaerandsen; dateIdentified: Feb-28-2020; **Event:** eventDate: Jun 25–Jul 19 2014; **Record Level:** institutionCode: CBG; collectionCode: BIOUG; ownerInstitutionCode: Centre for Biodiversity Genomics; basisOfRecord: PreservedSpecimen
- **Type status:** Paratype. **Occurrence:** catalogNumber: BIOUG06633-C03; recordNumber: L#12BIOBUS-1119; recordedBy: BIOBus 2012; individualCount: 1; sex: female; lifeStage: imago; preparations: Pinned, whole specimen in glycerine vial, terminalia detached, cleared and stored in separate glycerine vial; associatedMedia: http://www.boldsystems.org/index.php/MAS_DataRetrieval_OpenSpecimen?selectedrecordid=3435128; occurrenceID: urn:uuid:7e70d5b9-6a4d-425d-b96f-0e4c0245d88f; **Location:** country: Canada; stateProvince: ALberta; locality: Jasper National Park; decimalLatitude: 53.124; decimalLongitude: -117.775; geodeticDatum: WGS84; **Identification:** identifiedBy: Jostein Kjaerandsen; dateIdentified: Feb-28-2020; **Event:** eventDate: 17–25 Jul 2012; **Record Level:** institutionCode: CBG; collectionCode: BIOUG; ownerInstitutionCode: Centre for Biodiversity Genomics; basisOfRecord: PreservedSpecimen
- **Type status:** Paratype. **Occurrence:** catalogNumber: BIOUG21264-A06; recordNumber: GMP#03915; recordedBy: M.Otway; individualCount: 1; sex: female; lifeStage: imago; preparations: In 95% alcohol; associatedMedia: http://www.boldsystems.org/index.php/MAS_DataRetrieval_OpenSpecimen?selectedrecordid=5833545; occurrenceID: urn:uuid:04f350dd-49a0-4c09-a145-1d37c3e32544; **Location:** country: Canada; stateProvince: Saskatchewan; locality: Grasslands National Park; decimalLatitude: 49.001; decimalLongitude: -106.557; geodeticDatum: WGS84; **Identification:** identifiedBy: Jostein Kjaerandsen; dateIdentified: Feb-28-2020; **Event:** eventDate: Jun 29–Jul 17 2014; **Record Level:** institutionCode: CBG; collectionCode: BIOUG; ownerInstitutionCode: Centre for Biodiversity Genomics; basisOfRecord: PreservedSpecimen

#### Description

Male.(Figs 5, 7, 8, 11c, d, 13, *n* = 1 [holotype])

Colouration and most body characteristics (Fig. 5) as in *Coelosynapha loici* sp. n. and will not be repeated here.

Wings (Fig. 7a, b). Wing length 2.6 mm. Crossveins **sc–r** and **R_2+3_** absent (in holotype, Fig. 7b). Posterior fork longer, its stem forking opposite of anterior fork (Fig. 7a). Veins **M_4_** and **CuA** branching with acute angle and less divergent at base of fork. Sc with 6 macrotrichia on apical half.

Terminalia (Figs 11c, d, 13). Tergite 9 elongated, gradually tapering into acute apex, bearing two, strong, pale and 15 dark, apico-ventral, stout setae. Cerci and hypoproct/sternite 10 retracted well in under tergite 9. Gonocoxites with a deep ventral cleft. Gonocoxite with a hyaline, apico-mesal, long seta, almost as long as gonostylus. Gonostylus elongated, with three blunt, digitate projections apically (marked as a, b & c in Fig. 13a) , three lamellae subapically (marked as d, e & f in Fig. 13a, b) and two long bristles mesally (marked as g & h in Fig. 13b). Apical projection (a) finger-like, apically rounded, evenly broad and symmetrical. Subapical projection (b) narrow, black, apically rounded, glabrous. Basal projection (c) black, hollow, having reticulate surface pattern, glabrous except strong setae on a small projection laterally, apically rounded. Lateral lamella (d) subrectangular, high and narrow, covered with setae. Ventral lamella (e) forming a blunt ridge with row of 10 acute, short setae. Ventromesal lamella inconspicuous fold without specialised setae.

Female. (*n* = 4)

Colouration as for male.

Wings (Fig. 7c, d). Wing length 2.9 mm (*n* = 1). Venation in radial sector variable. One (SSJAD4978-13) of four females with crossvein sc–r present (Fig. 7d), two females with **R_2+3_** present and forming tiny cell, one female without **R_2+3_**. **Sc** with 3–6 macrotrichia on apical half, setation otherwise as for male.

Terminalia (Fig. 8) as described for genus.

#### Etymology

*Coelosynapha heberti* sp. n. is named in honour of Paul D. N. Hebert, "the father" of DNA barcoding who also led the project of barcoding the insects of Canada (Hebert et al. 2016) from which the new species was located through its barcode similarity with *Coelosynapha loici* sp. n. It is further an expression of the importance of DNA barcoding in the new integrative science of taxonomy.

#### Distribution

The new species has a wide Nearctic range across Canada (Fig. 2).

## Identification Keys

### Section of the generic key to separate *Coelosynapha* and *Coelosia*

**Table:** 

1	Point of furcation of posterior fork (veins **M_4_** and **CuA**) distinctly beyond point of furcation of anterior fork (veins **M_1_** and **M_2_**)	2
–	Point of furcation of posterior fork (veins **M_4_** and **CuA**) before, below or slightly beyond point of anterior fork (veins **M_1_** and **M_2_**)	Couplet 69 in the key by Søli et al. (2000)
2	Stem of anterior fork more than 2x longer than crossvein **r–m**, **R_2+3_** present or absent, crossvein **sc–r** present or absent	* Coelosynapha * **gen. n.**
–	Stem of anterior fork at most slightly longer than crossvein **r–m**, **R_2+3_** always absent, Crossvein **sc–r** always absent	*Coelosia* Winnertz, 1864

Couplet 1 corresponds to the couplet 68 in the key by Søli et al. (2000)

## Discussion

The enormous success of DNA barcoding now has accumulated a substantial amount of sequenced insects on BOLD, very useful for new and integrative taxonomic studies. More than 65,000 specimens belonging to the family Mycetophilidae have been successfully sequenced and, of them, some 10,000 are assigned to the subfamily Gnoristinae. Some 1100 identified Mycetophilidae species have public barcodes although more than 2400 different BINs are assigned, thus indicating that the majority of the species still remains unidentified beyond the (sub)family level on BOLD. A weakness with the BOLD initiative may be that several of the typically well-funded, large scale DNA barcoding projects, undertaken so far, did not have a focus on, nor adequate resources allocated to, securing high quality morphological identification of the vouchers for the accumulated barcodes. Unfortunately, this critical endeavour of the BOLD archive is largely left to the under-funded and scarce taxonomic expertise to engage in "*post-sequence*" at will.

In the Nordic region, however, strong ties between The Norwegian and Swedish Biodiversity Information Centres, including their Taxonomy Initiatives and NorBOL and FinBOL, are ensuring that the best taxonomic expertise is building up the reference library of the local fauna on the BOLD archive. Hence, the vast majority of some 6500 DNA barcoded fungus gnats (Sciaroidea) from the Nordic region have been identified to species level upon submission and the reference library is profoundly and repeatedly quality-checked and curated after barcodes and BINs are assigned. This has resulted in a high quality reference library, now covering about 90% of the known fauna and more than 100 additional species considered to be new to science (Kjærandsen 2020, Kjærandsen 2017). Hence, when the sequences of *Coelosynapha* gen. n. most closely resembles exotic, unidentified species of Mycetophilidae from South Australia and Costa Rica (Fig. 14a), this is a strong indication of a genus not previously known from the region. When we restrict the dataset to the 6500 sequences, representing nearly all genera of Nordic Sciaroidea, *Coelosynapha* gen. n. is most closely aligned with the genus *Palaeodocosia* Meunier, 1904 (Fig. 14b), while species of both *Synapha* and *Coelosia* appear more distant.

*Coelosynapha loici* sp. n. is assigned to the BIN BOLD:ADD0785, consisting of 10 members with a maximum within-species genetic distance of 0.72%. *Coelosynapha heberti* sp. n. with five members assigned to BIN BOLD:ACI5160 likewise has a maximum within-species genetic distance of 0.72%. The reciprocal nearest-neighbour distance between the two is 8.27–8.66% (depending on direction). A barcode gap analysis of the distance between *Coelosynapha loici* sp. n. and close genera reveals the closest *Coelosia* being *Coelosia truncata* Lundström, 1909 at 13.67%, the closest *Synapha* being *Synapha fasciata* Meigen, 1818 at 15.36% and the sole determined *Austrosynapha* sp. on BOLD (sp. JSGS1, mined from GenBank) at 16.79% distance.

The variation seen in the radial sector of the wing in species of *Coelosynapha* gen. n. is not unique. Several Gnoristinae genera show variation in exactly this character, including *Grzegorzekia* Edwards, 1941, *Speolepta* Edwards, 1925, *Synapha* and *Syntemna* Winnertz, 1864, to a point where the instability is almost a characteristic of parts of the subfamily. None of these genera, however, appears to be very closely related to *Coelosynapha* gen. n. as revealed by COI haplotype similarities.

There are indications that both *Synapha and Austrosynapha*, as presently defined, are polyphyletic genera in need of revision, but it is beyond the scope of this paper to address these issues pending a more thorough revision of the entire subfamily. Here we have added another genus that, if included in either of them or in *Coelosia*, would likely render them even more para- or polyphyletic. We hope that describing *Coelosynapha* gen. n. will give new insights and inspire further phylogenetic studies of the fascinating, but intriguing subfamily Gnoristinae.

## Supplementary Material

28E14CF4-76EE-5317-A906-34EDA1844A22 10.3897/BDJ.8.e54834.suppl1 Supplementary material 1 DNA barcodes of specimens of *Coelosynapha* gen. n. downloaded from BOLD Data type DNA barcodes in fasta format Brief description The headings are listed with process ID on BOLD, species name, specimen code, country, state/region, mitochondrial gene region, BIN and, when available, GenBank index number. File: oo_412056.txt https://binary.pensoft.net/file/412056 Kjærandsen, J., Polevoi, A. & Salmela, J.

XML Treatment for Coelosynapha

XML Treatment for Coelosynapha loici

XML Treatment for Coelosynapha heberti

## Figures and Tables

**Figure 3a.**
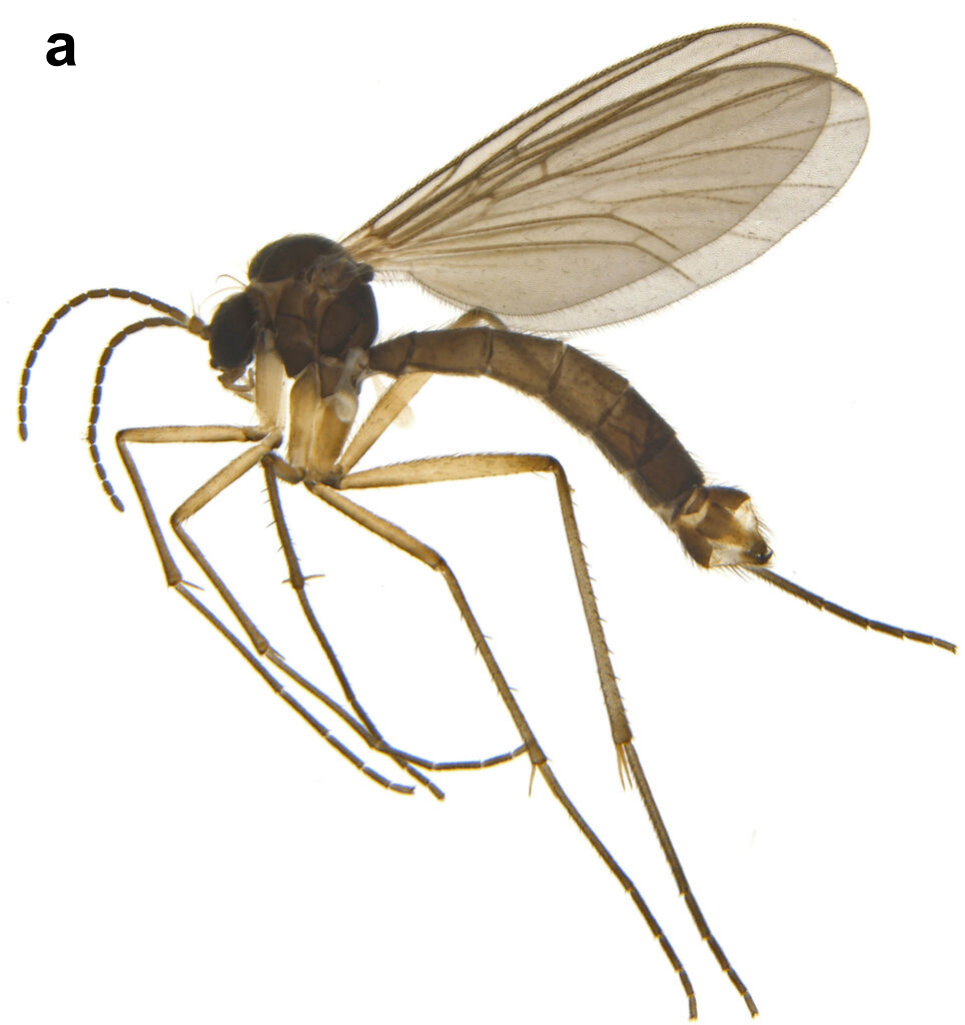
Habitus while still in 80% ethanol.

**Figure 3b.**
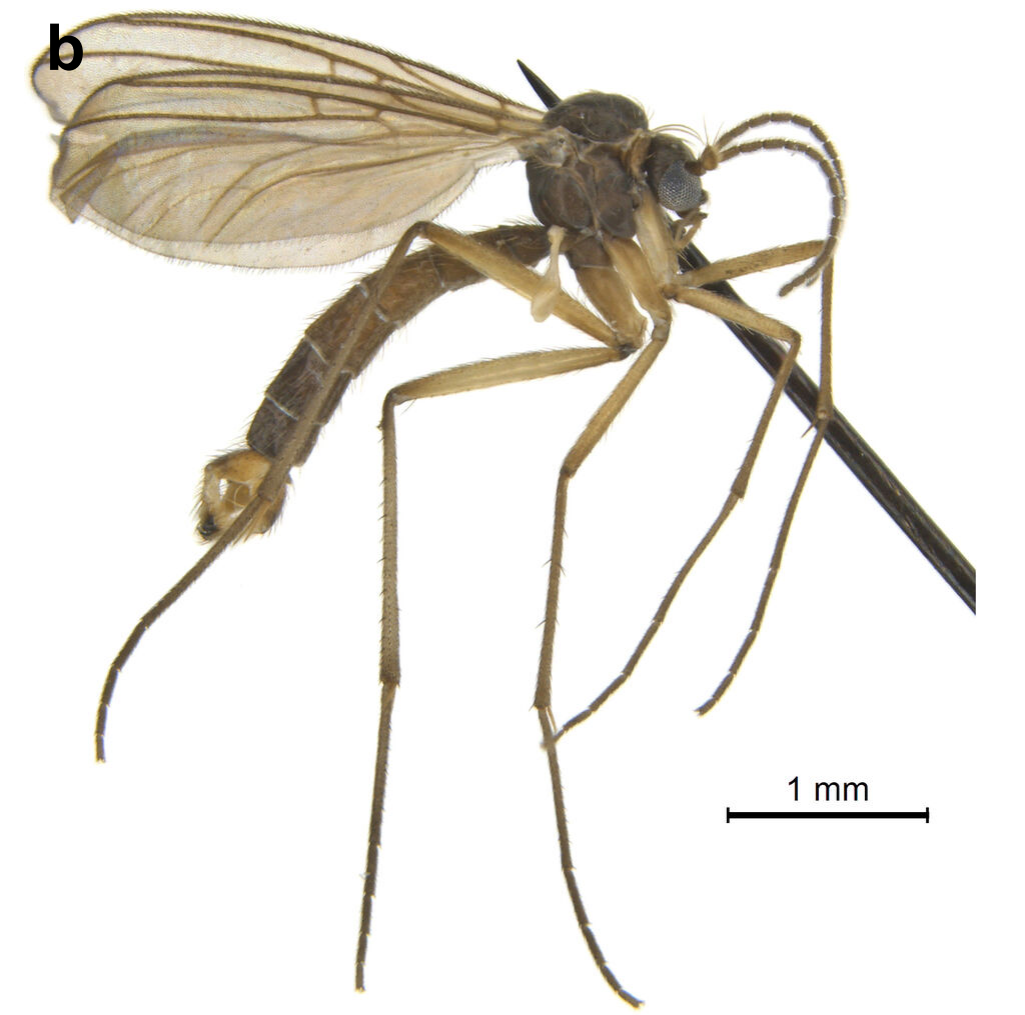
Habitus after drying and pinning (glued to a micropin) by HMDS baths from ethanol.

**Figure 4a.**
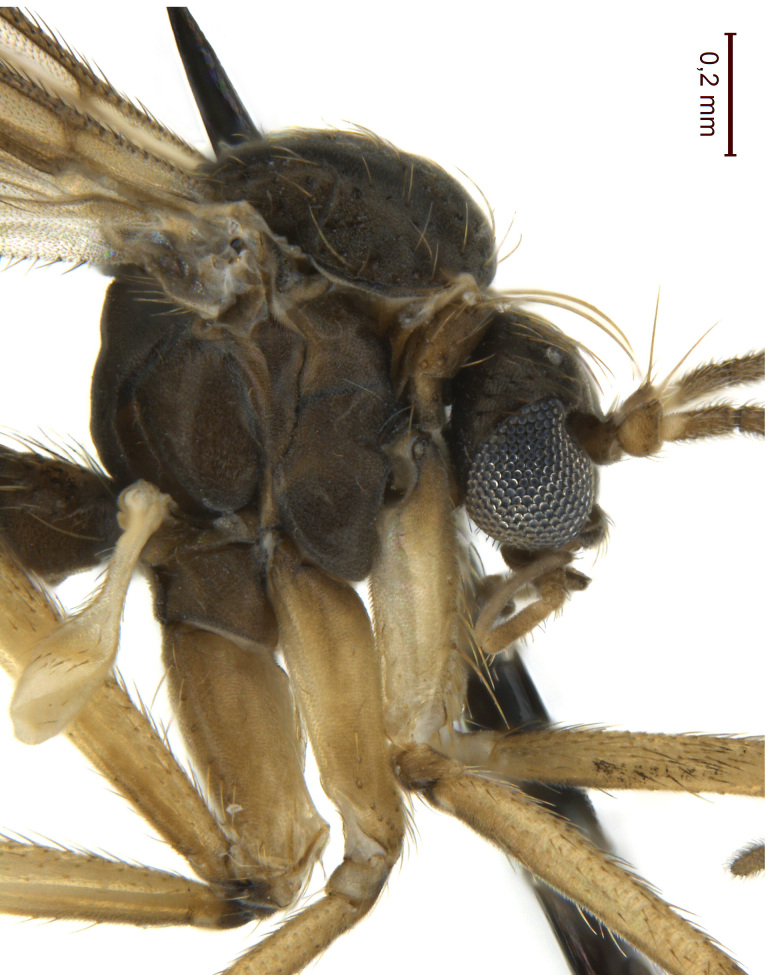
Thorax, lateral view.

**Figure 4b.**
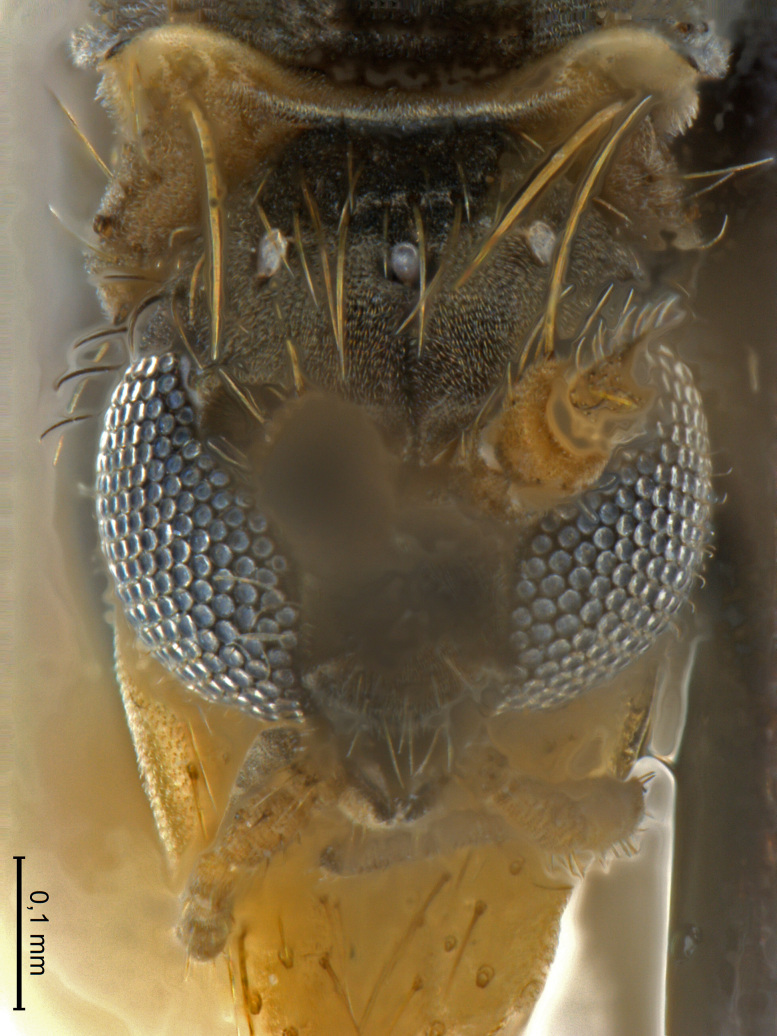
Head, frontal view.

**Figure 4c.**
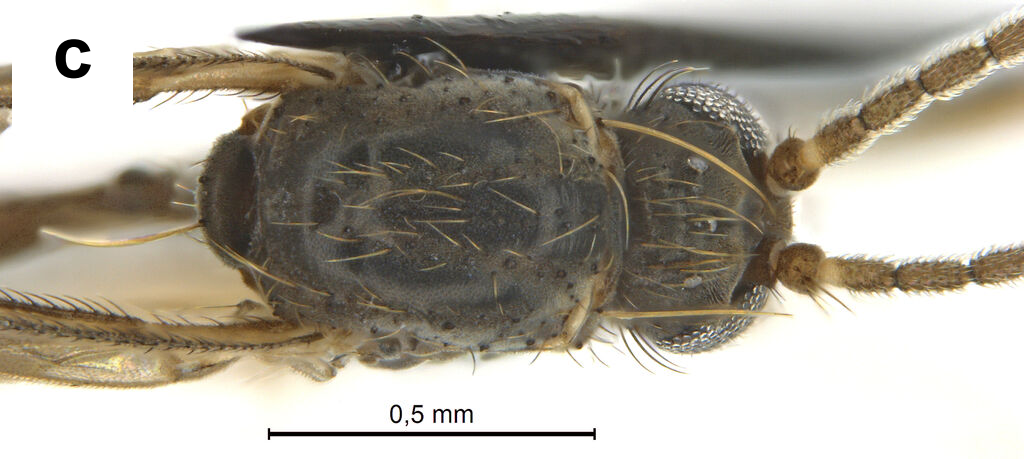
Head and thorax, dorsal view.

**Figure 6a.**
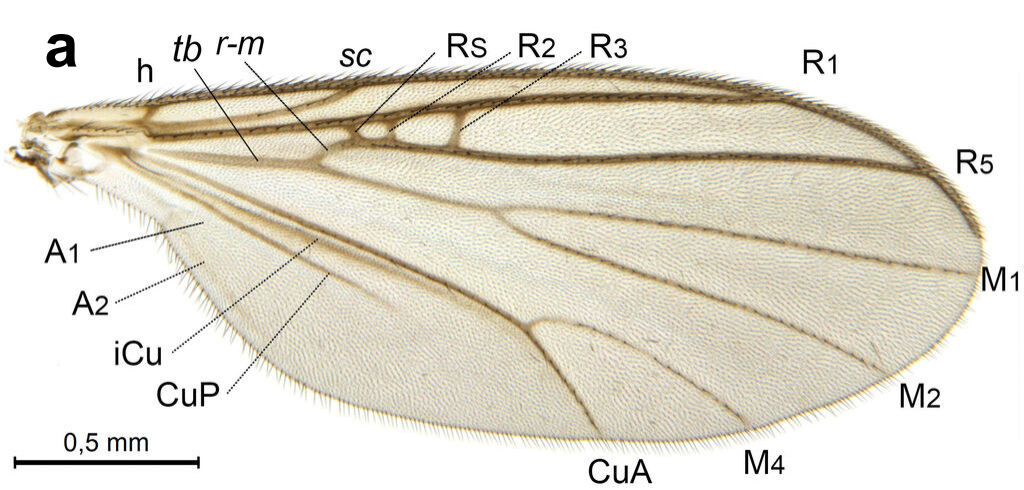
Paratype, male 1, whole wing

**Figure 6b.**
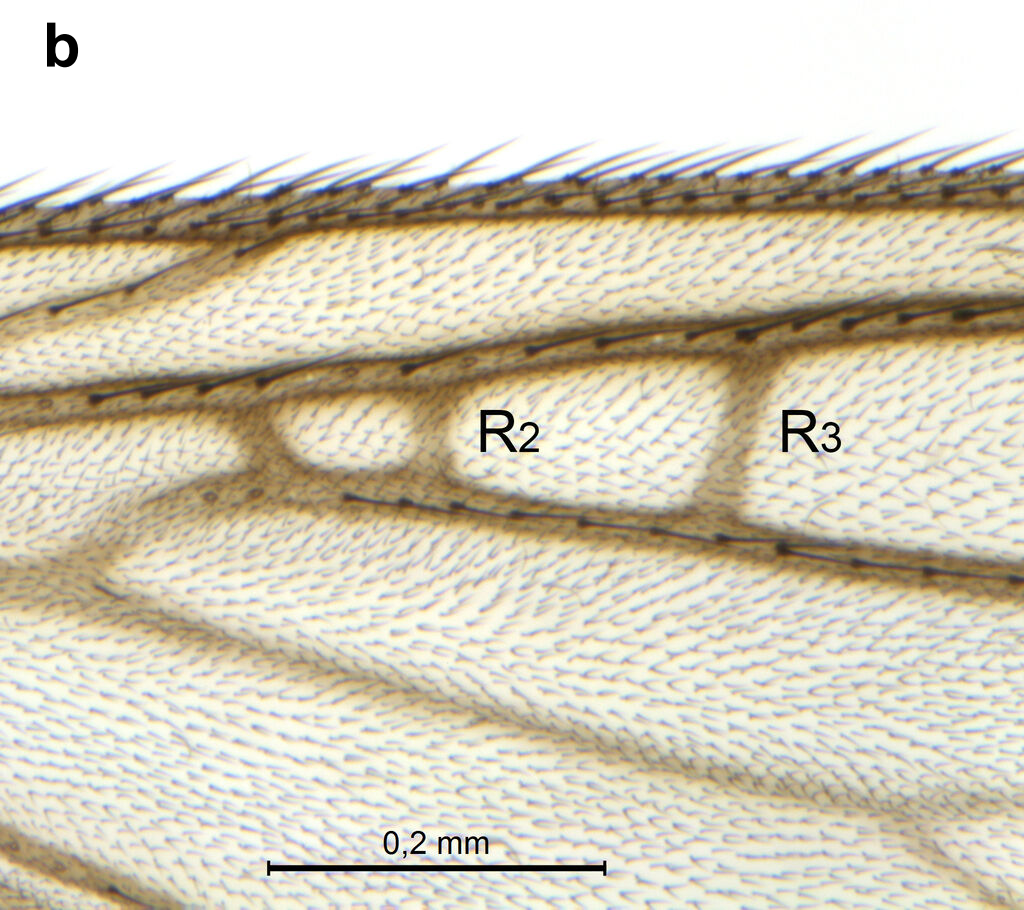
Paratype, male 1, details of radial sector.

**Figure 6c.**
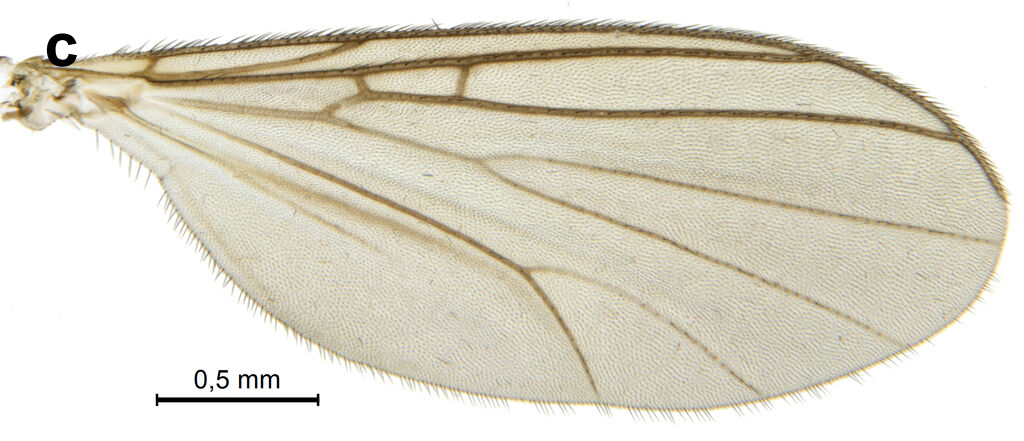
Paratype, male 2, whole wing

**Figure 6d.**
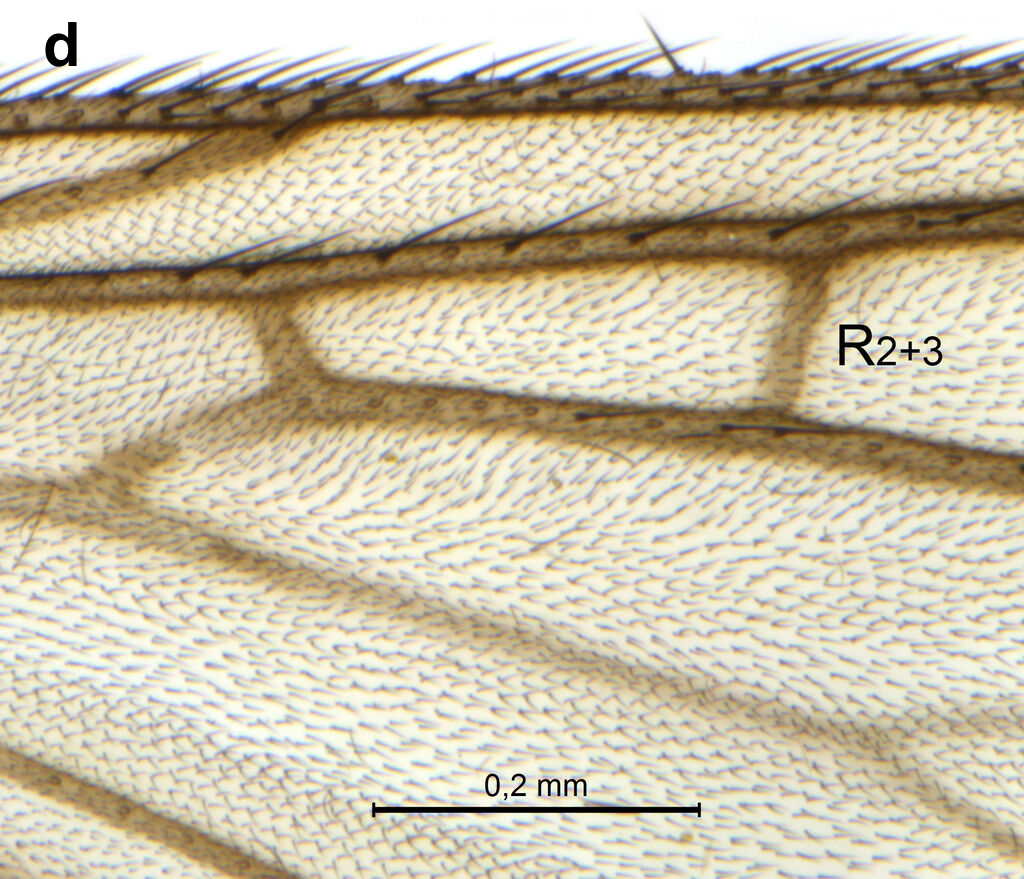
Paratype, male 2, details of radial sector.

**Figure 6e.**
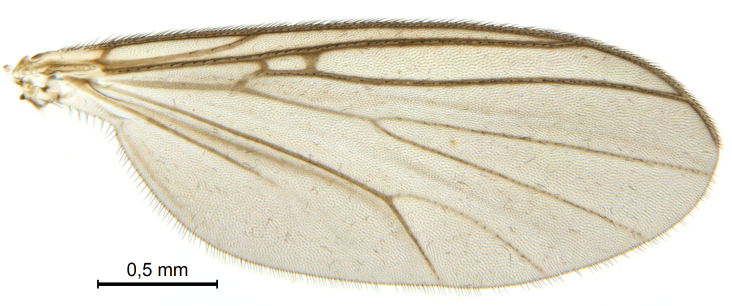
Paratype, male 3, whole wing

**Figure 6f.**
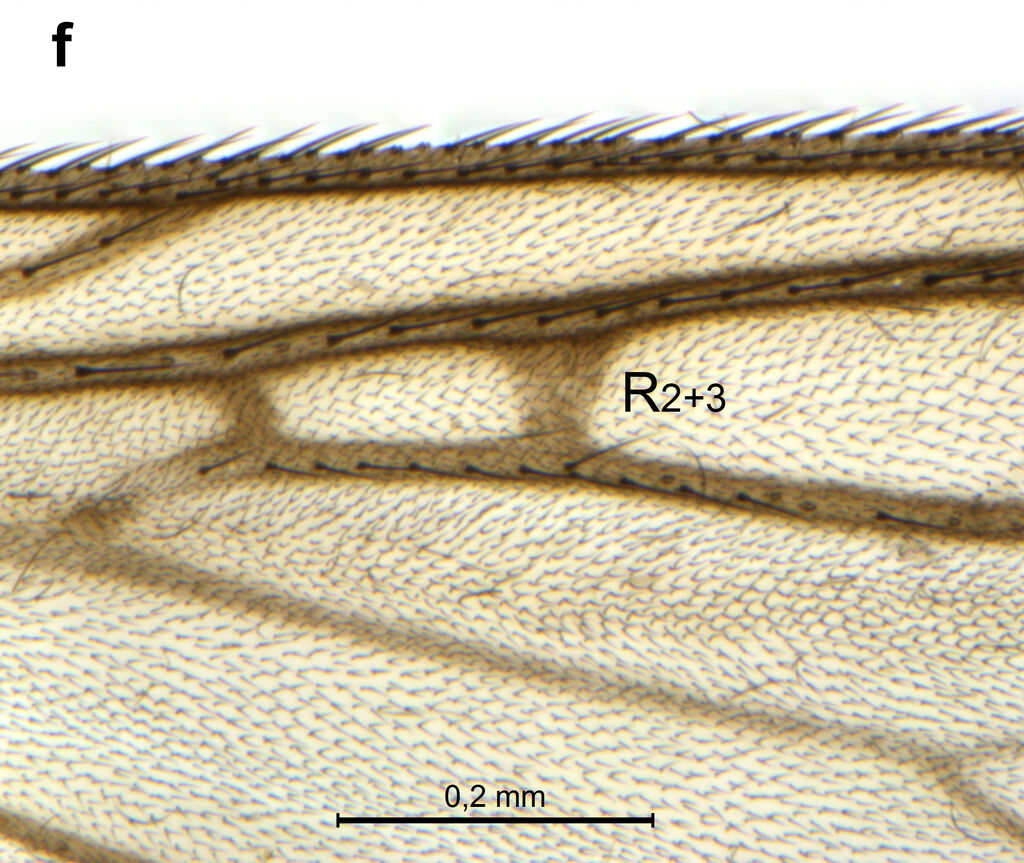
Paratype, male 3, details of radial sector.

**Figure 7a.**
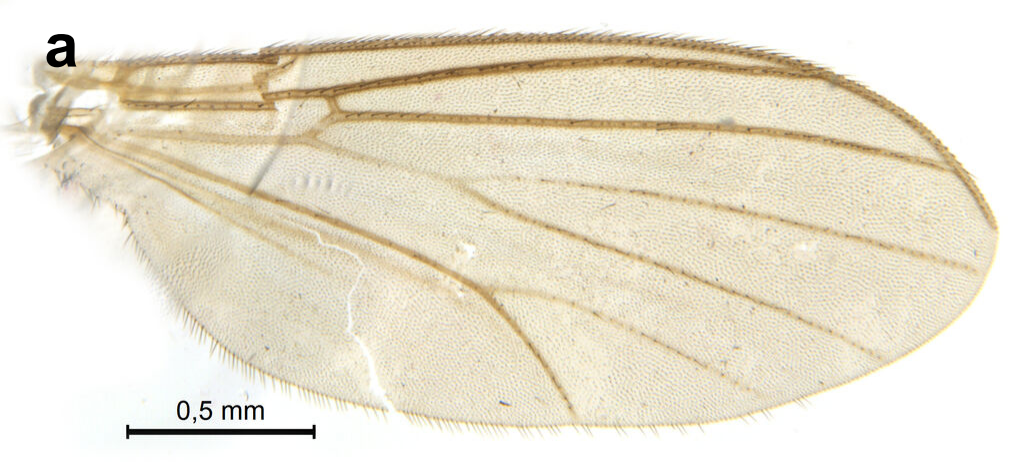
Holotype, male, whole wing.

**Figure 7b.**
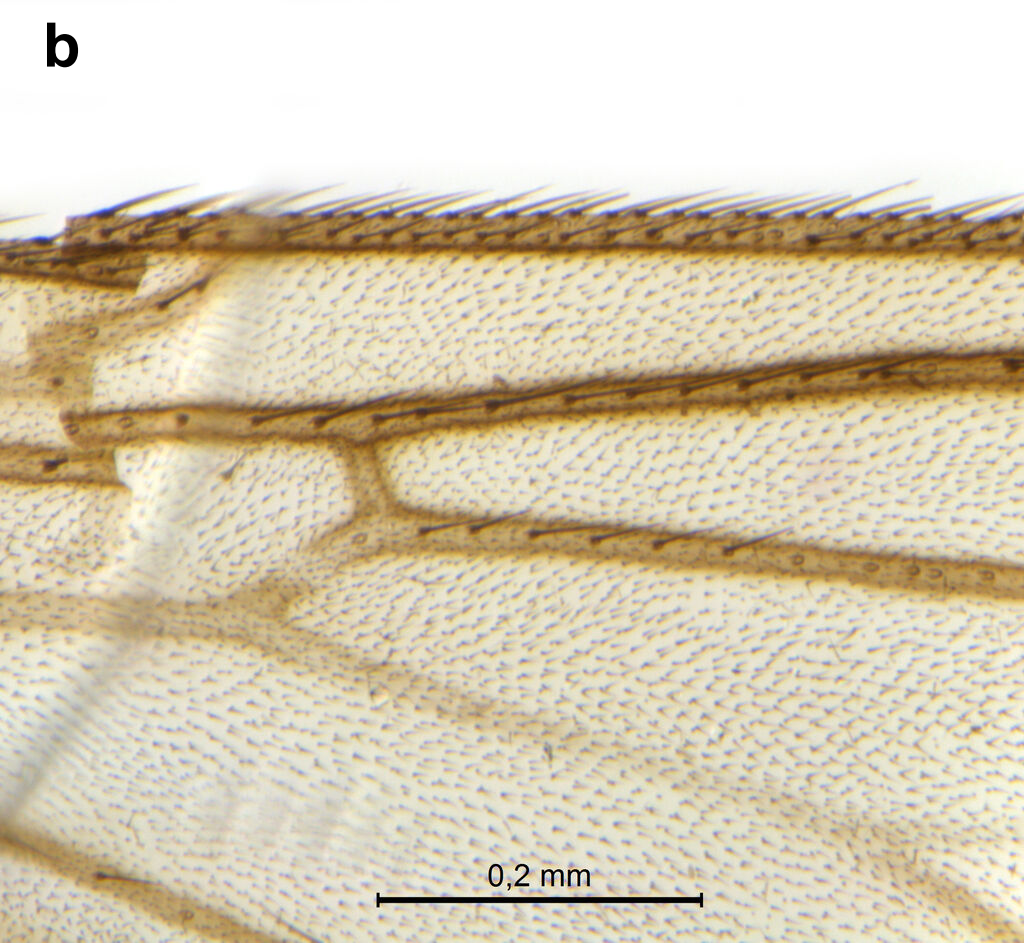
Holotype, male, details of radial sector.

**Figure 7c.**
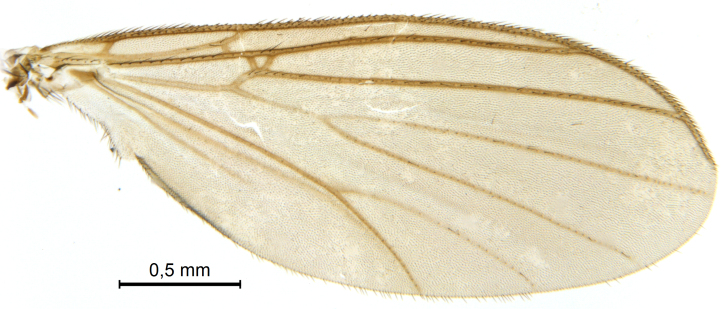
Paratype, female, whole wing.

**Figure 7d.**
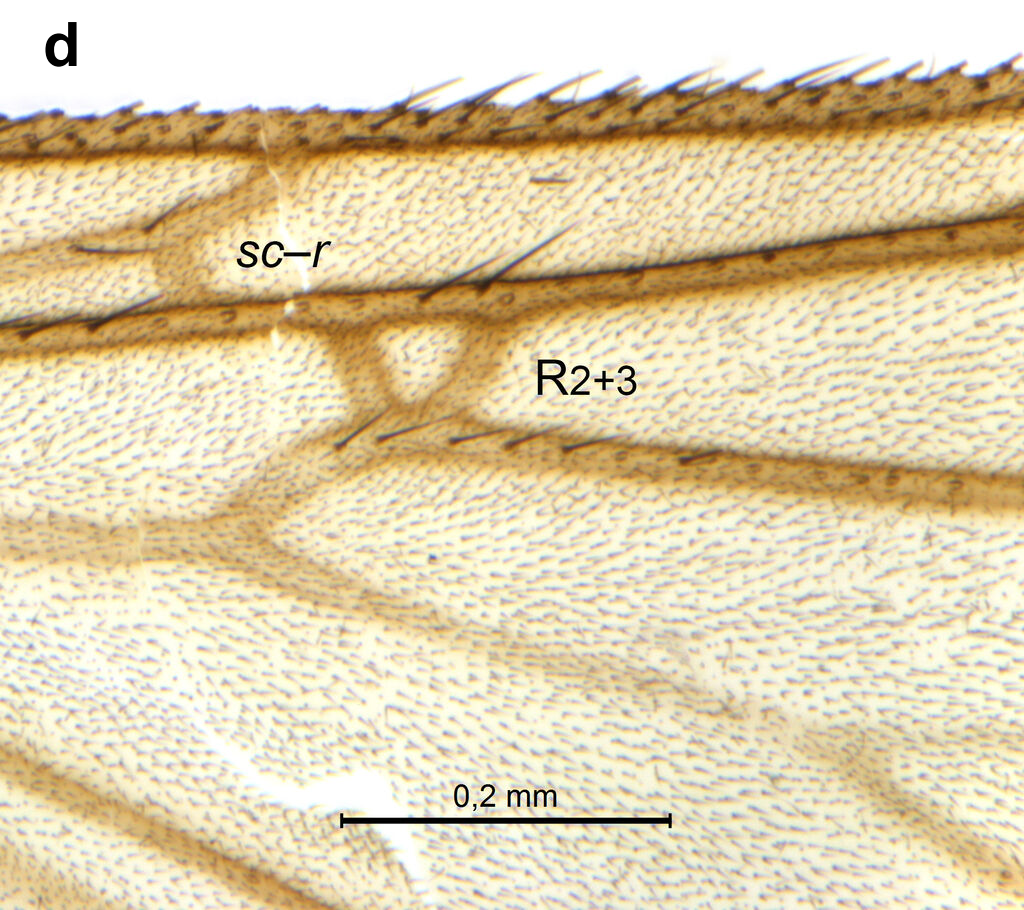
Paratype, female, details of radial sector.

**Figure 9a.**
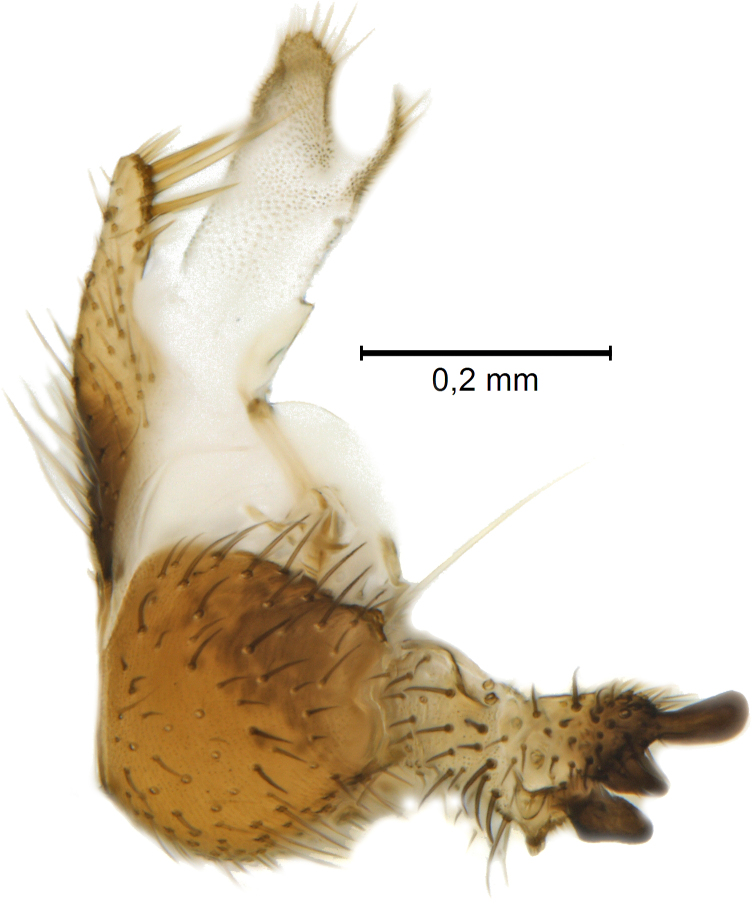
Stacked image, lateral view.

**Figure 9b.**
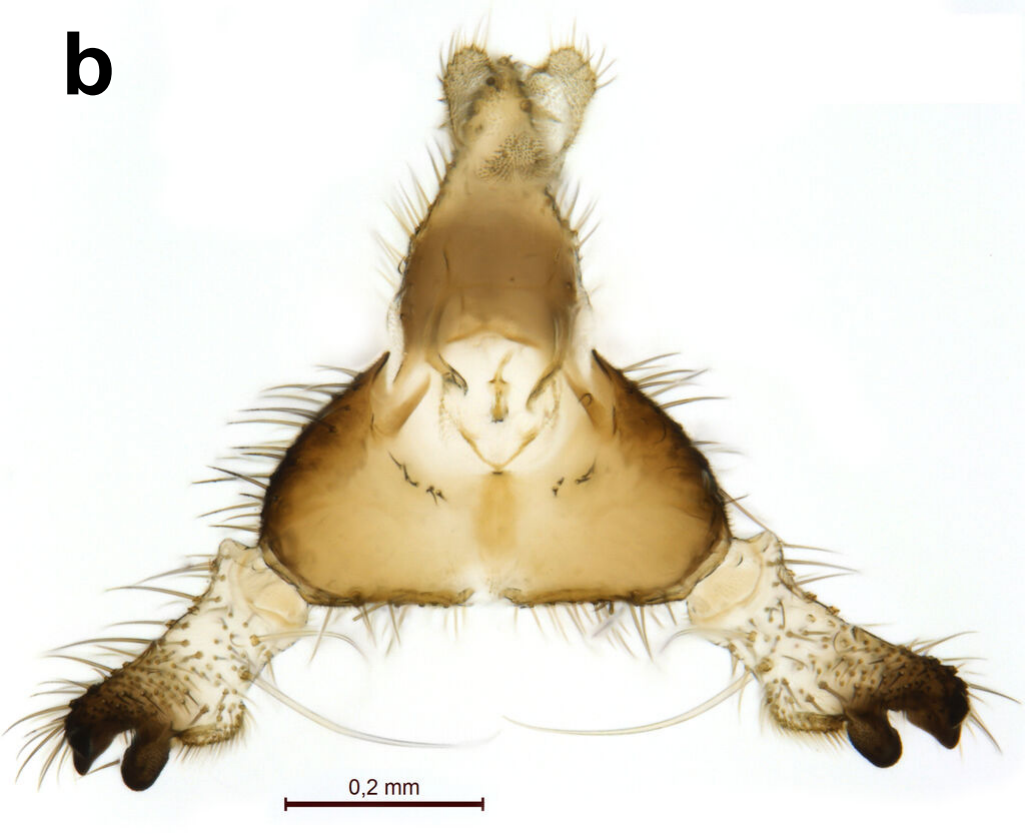
Stacked image, caudal view.

**Figure 9c.**
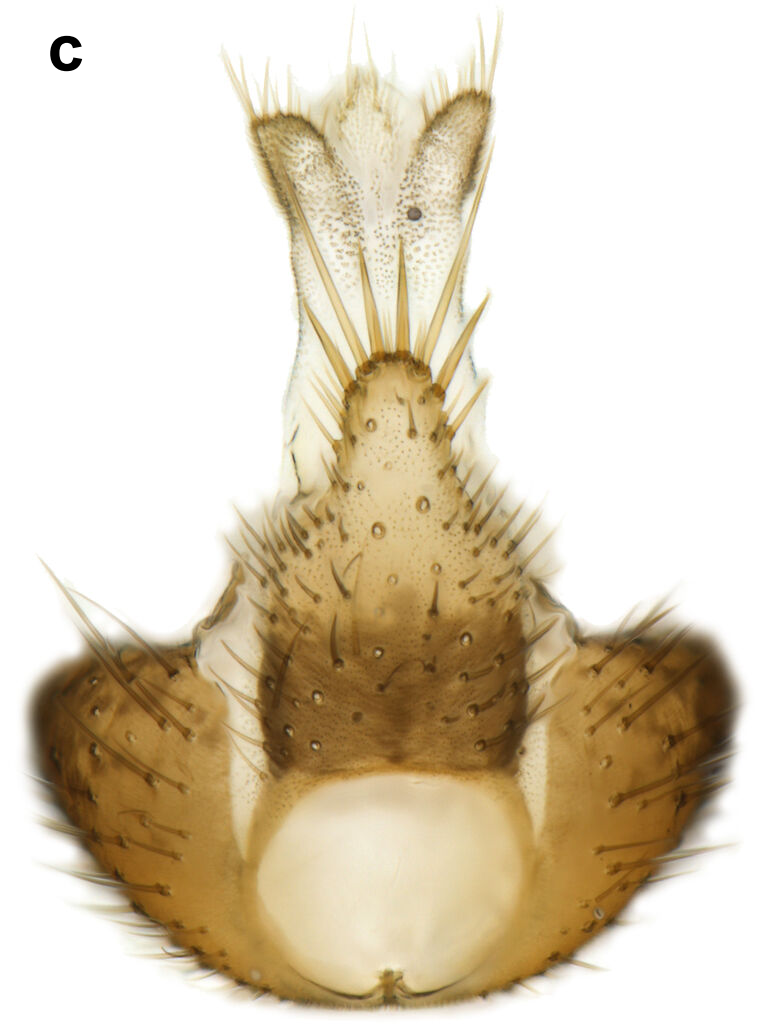
Stacked image, dorsal view.

**Figure 10a.**
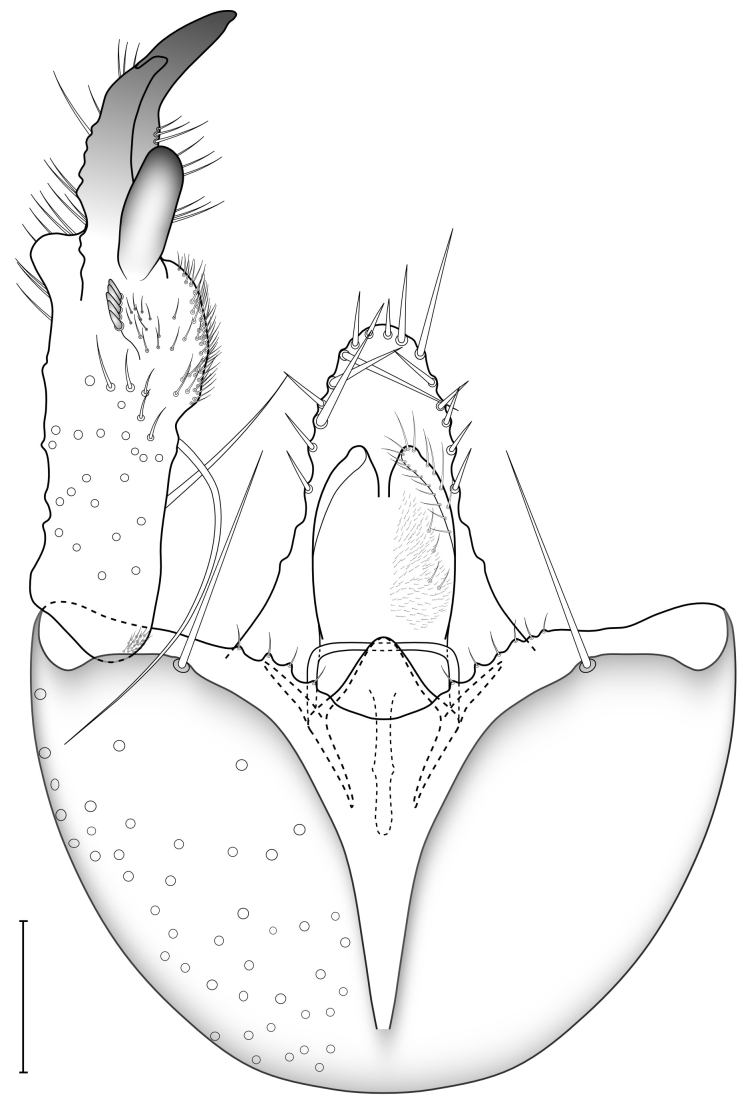
Terminalia, ventral view.

**Figure 10b.**
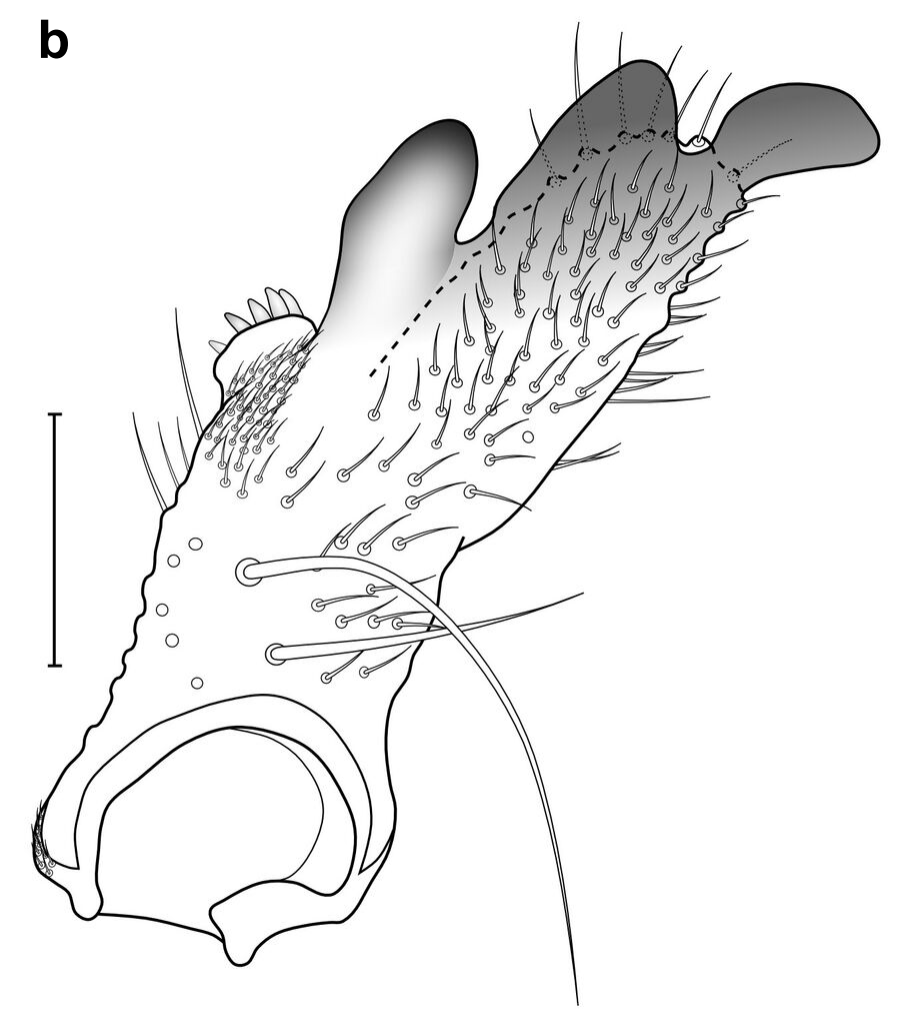
Gonostylus, internal view.

**Figure 10c.**
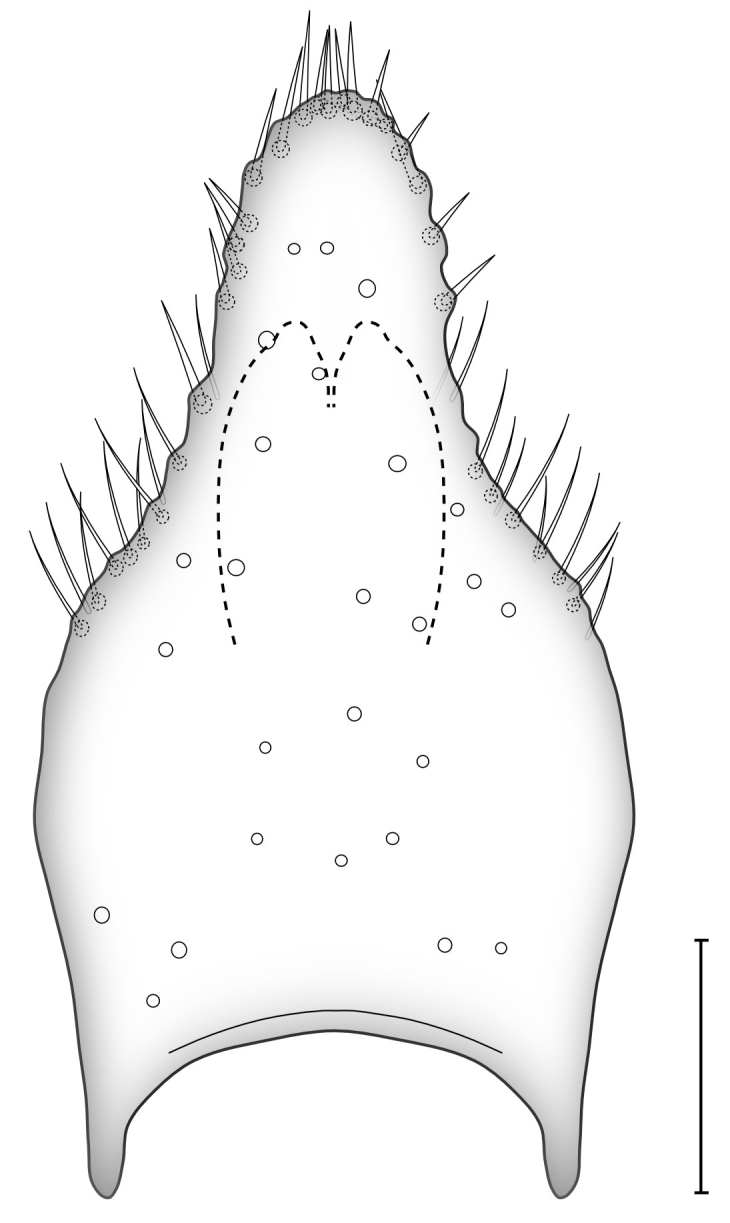
Tergite 9, dorsal view.

**Figure 11a.**
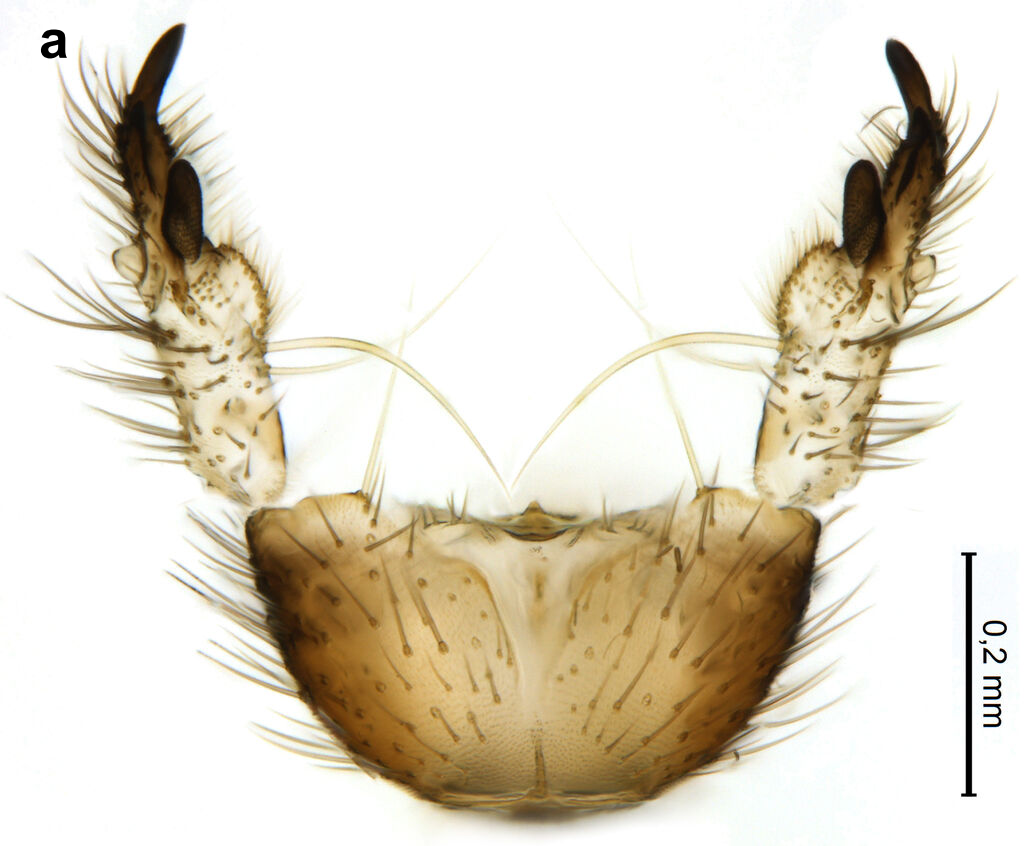
*Coelosynapha loici* sp. n., ventral view.

**Figure 11b.**
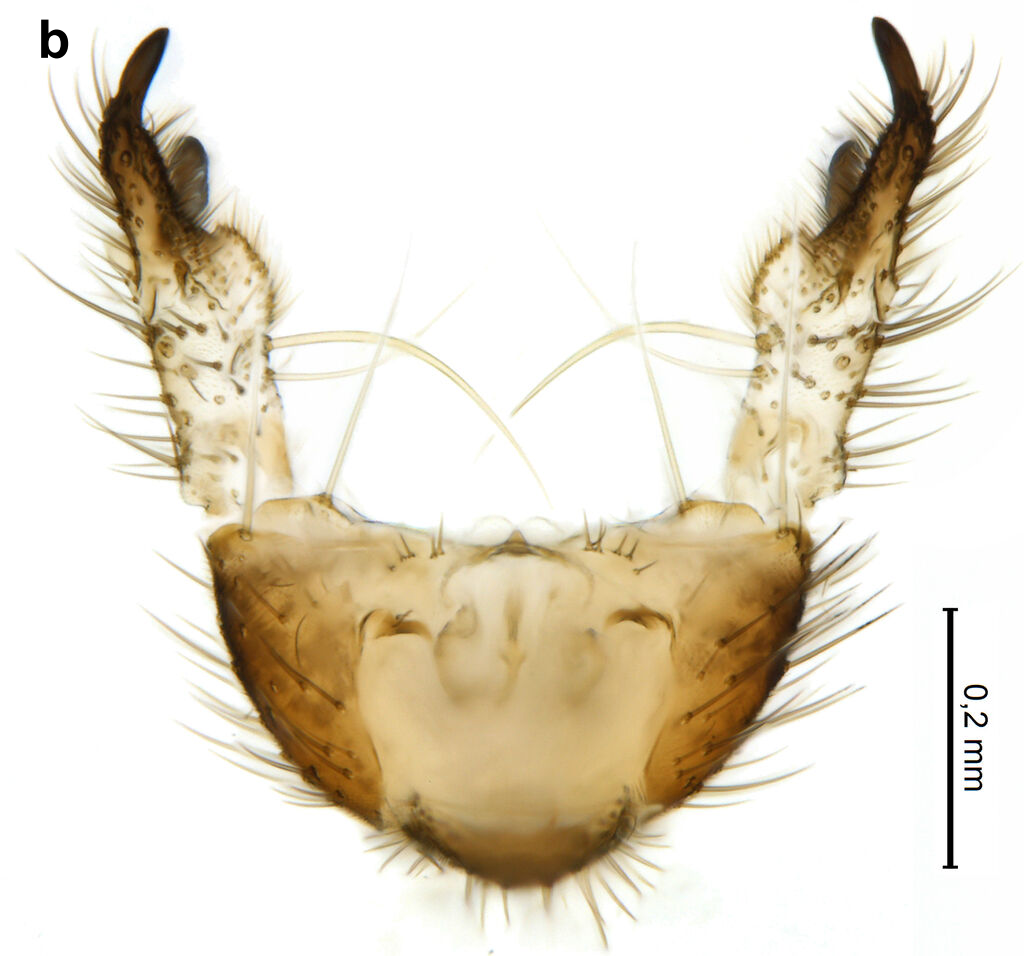
*Coelosynapha loici* sp. n., dorsal view.

**Figure 11c.**
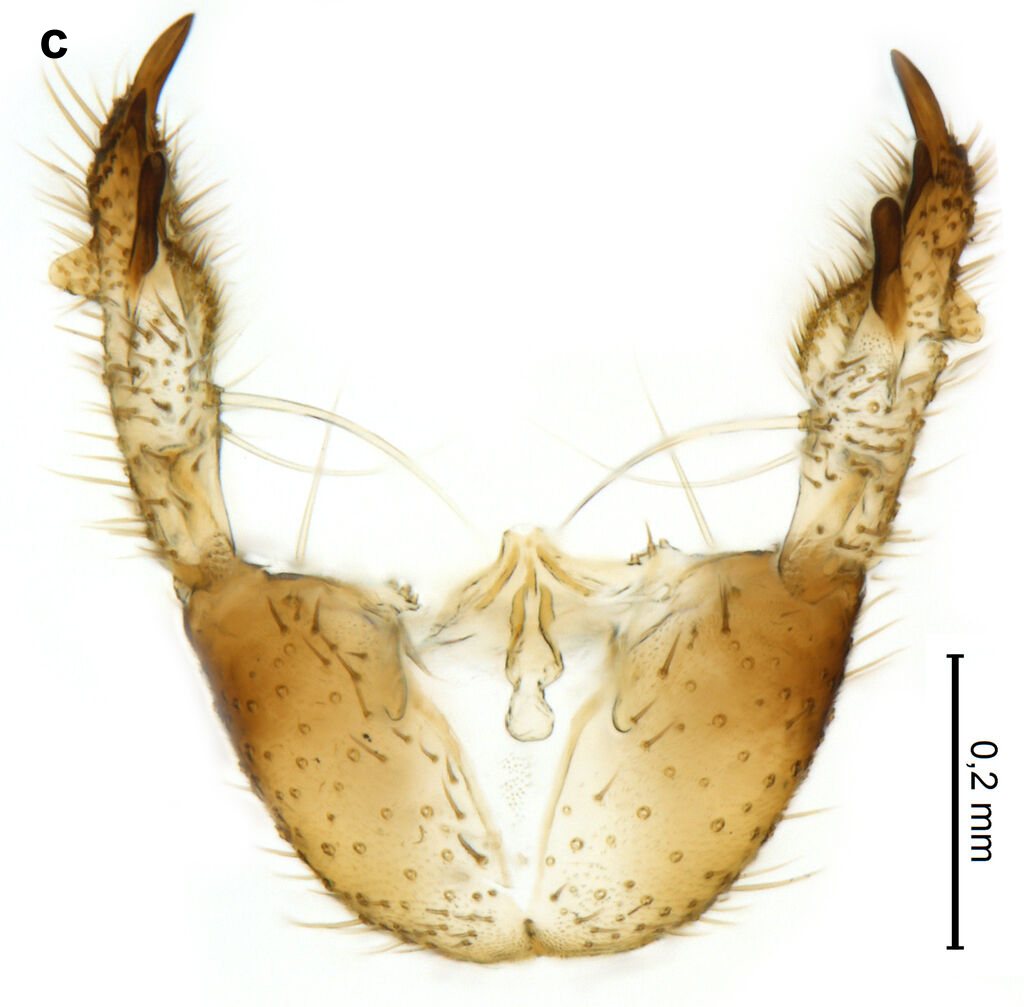
*Coelosynapha heberti* sp. n., ventral view.

**Figure 11d.**
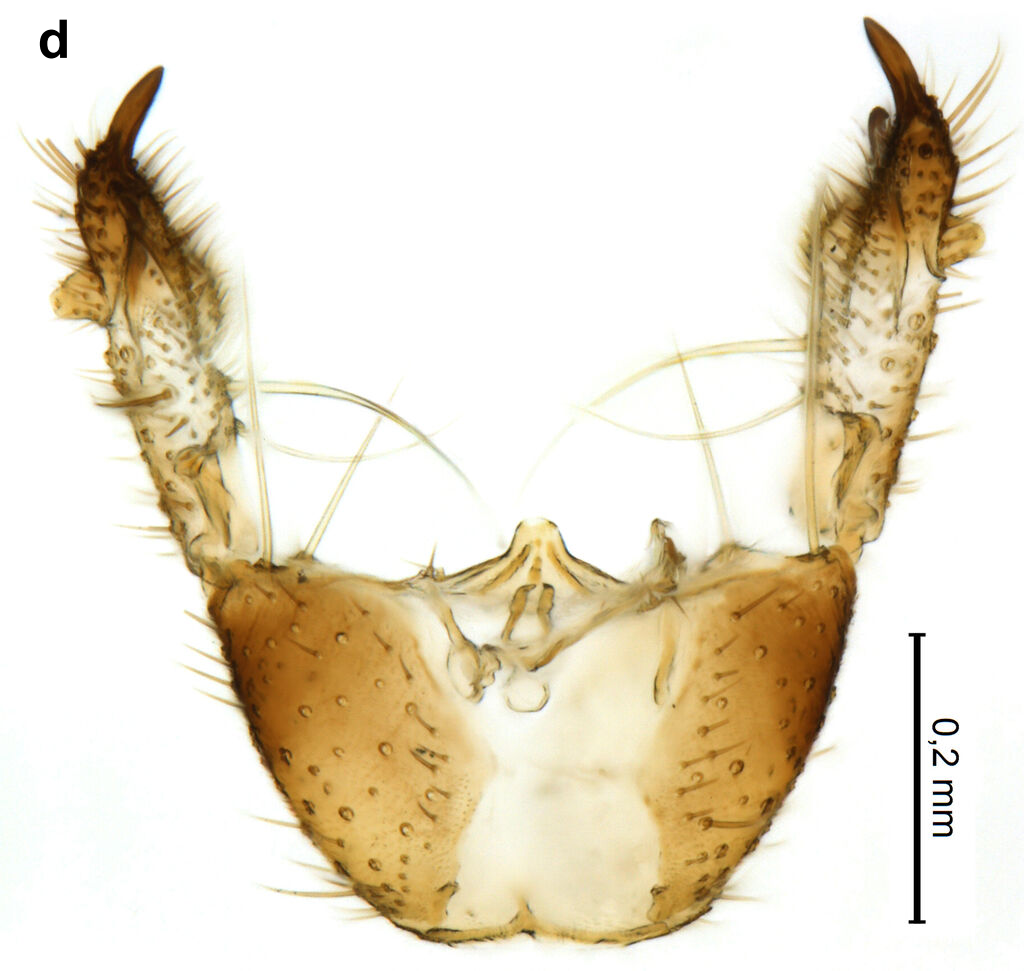
*Coelosynapha heberti* sp. n., dorsal view, tergal parts removed.

**Figure 12a.**
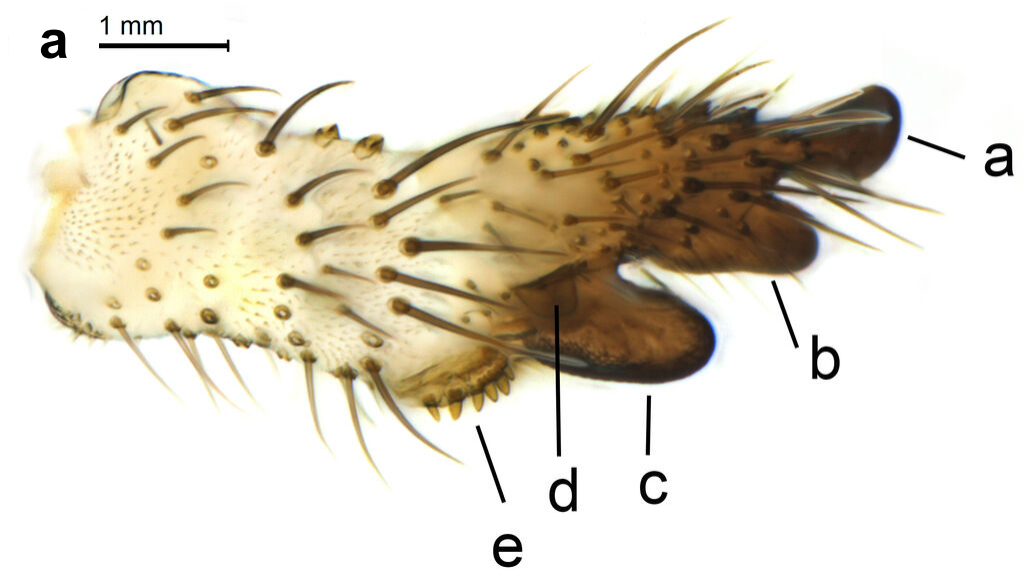
Gonostylus, external view.

**Figure 12b.**
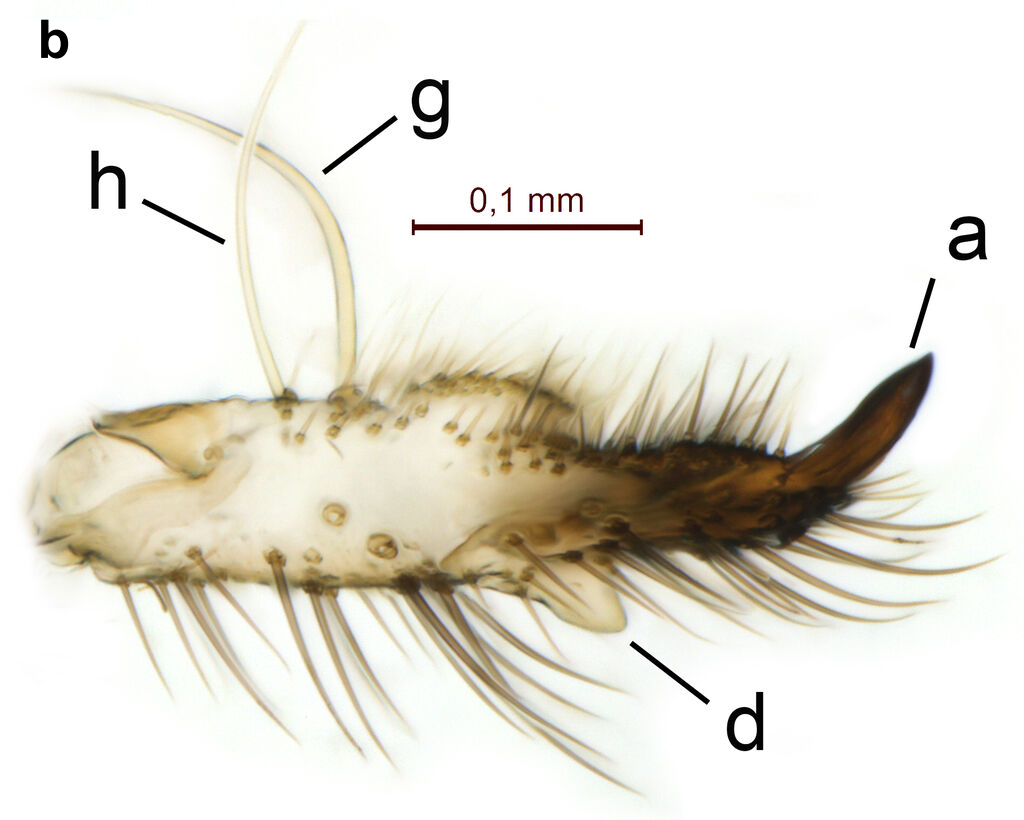
Gonostylus, dorsal view.

**Figure 12c.**
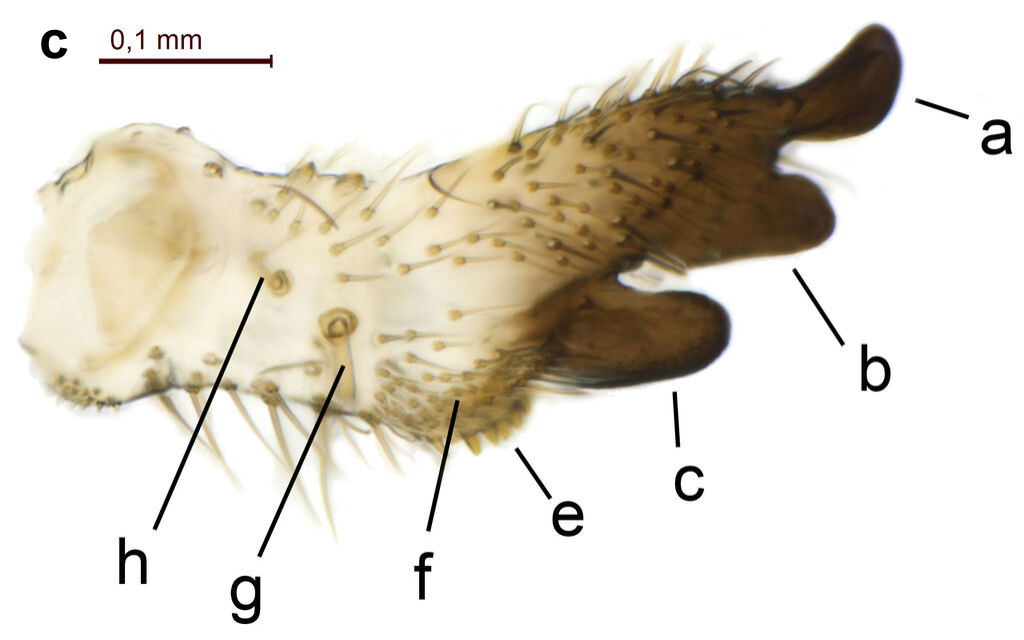
Gonostylus, internal view.

**Figure 12d.**
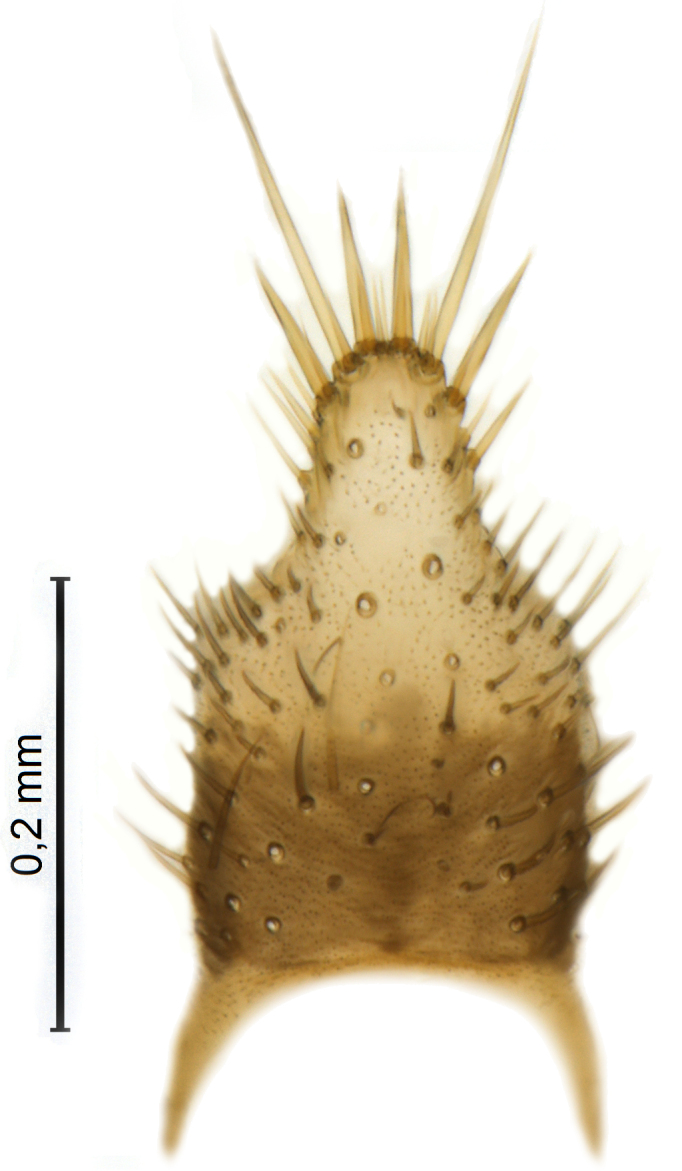
Tergite 9, dorsal view.

**Figure 13a.**
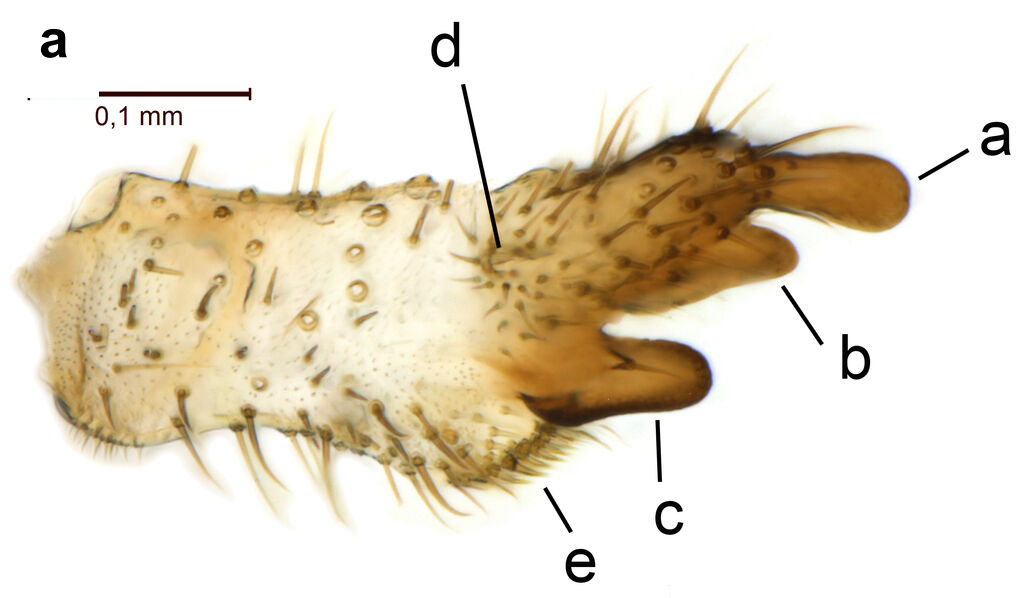
Gonostylus, external view.

**Figure 13b.**
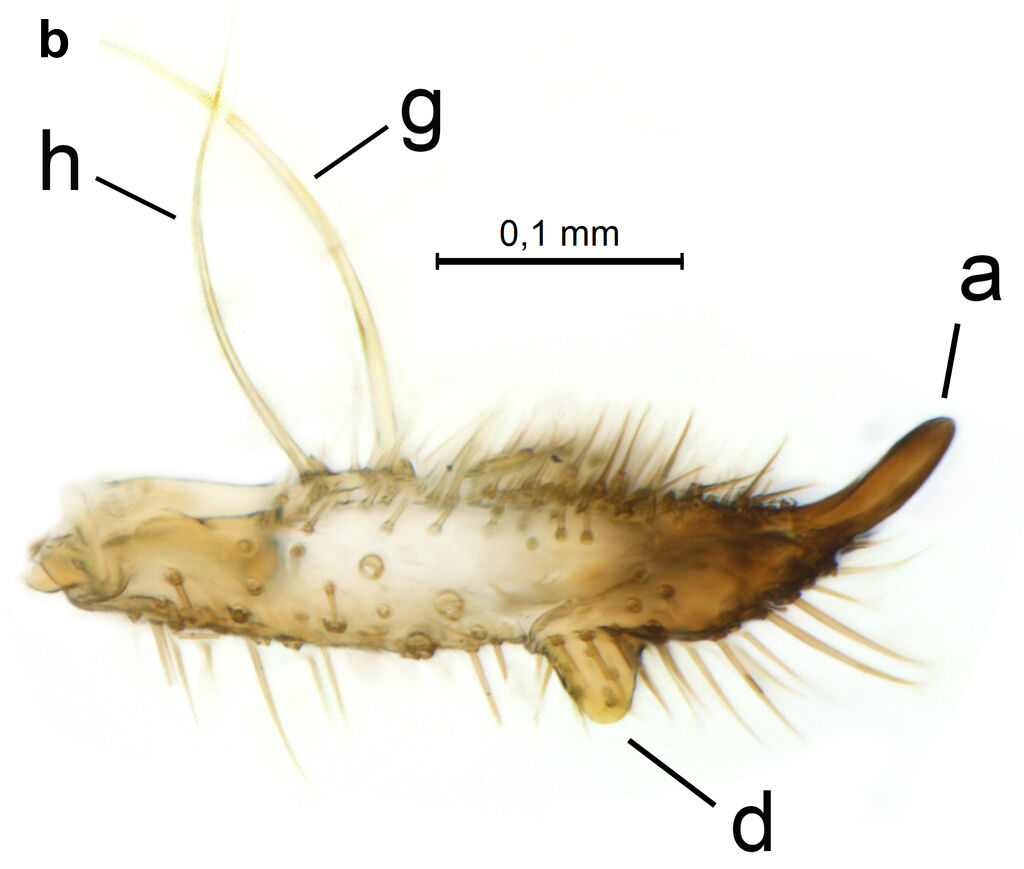
Gonostylus, dorsal view.

**Figure 13c.**
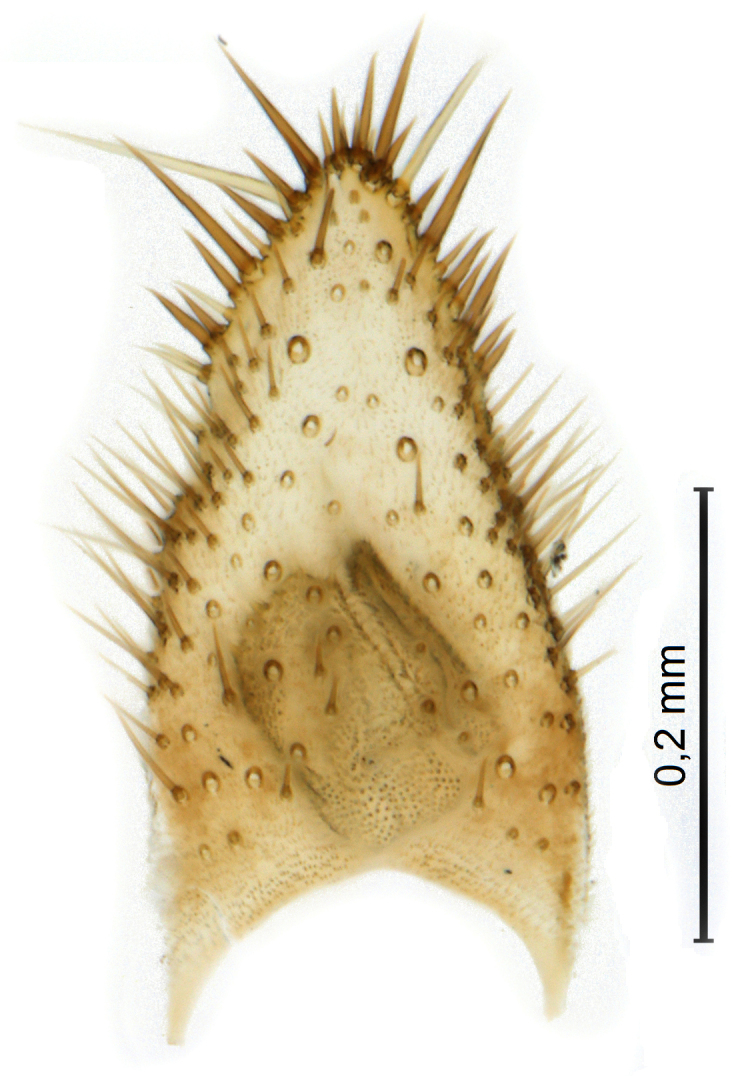
Tergite 9, dorsal view.

**Figure 14a.**
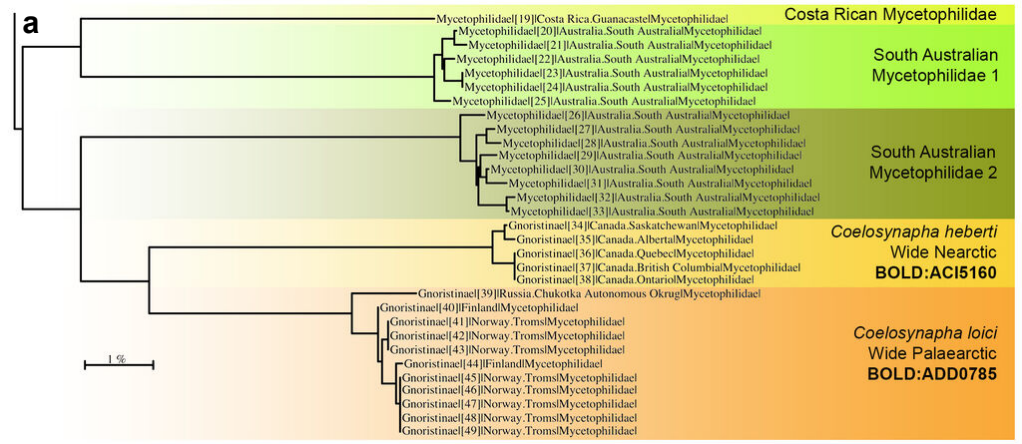
Taxa of the new genus *Coelosynapha* gen. n. and their closest hits on the entire BOLD database as of 10 March 2020. Three unidentified taxa from South Australia and Costa Rica display the closest haplotype similarity to the new genus.

**Figure 14b.**
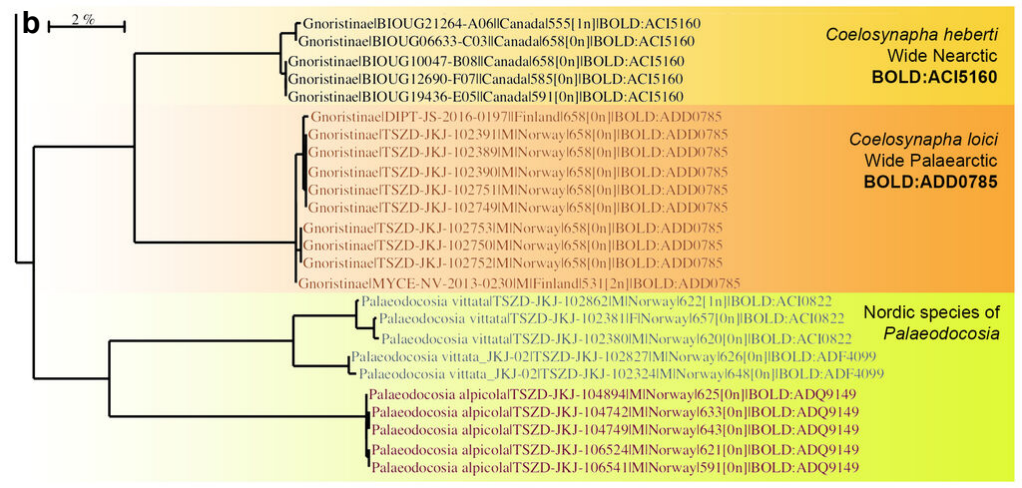
The taxa of the new genus *Coelosynapha* gen. n. in a dataset of 6500 sequences of Nordic Sciaroidea clearly separates the two species in distinct BINs. Locally in the Nordic Region, the genus *Palaeodocosia* Meunier, 1904 show highest similarity with *Coelosynapha*, while species of both *Synapha* and *Coelosia* are more distant.
